# *In Vivo* Fiber Optic Raman Spectroscopy of Muscle in Preclinical Models of Amyotrophic Lateral Sclerosis and Duchenne Muscular Dystrophy

**DOI:** 10.1021/acschemneuro.0c00794

**Published:** 2021-05-05

**Authors:** Maria Plesia, Oliver A. Stevens, Gavin R. Lloyd, Catherine A. Kendall, Ian Coldicott, Aneurin J. Kennerley, Gaynor Miller, Pamela J. Shaw, Richard J. Mead, John C. C. Day, James J. P. Alix

**Affiliations:** † Sheffield Institute for Translational Neuroscience, University of Sheffield, Sheffield S10 2HQ, UK; ‡ Interface Analysis Centre, School of Physics, University of Bristol, Bristol BS8 1TL, UK; § Phenome Centre Birmingham, University of Birmingham, Birmingham B15 2TT, UK; ∥ Biophotonics Research Unit, Gloucestershire Hospitals NHS Foundation Trust, Gloucester GL1 3NN, UK; ⊥ Department of Chemistry, University of York, York YO10 5DD, UK; # Department of Oncology and Metabolism, University of Sheffield, Sheffield S10 2RX, UK; ○ Cross-Faculty Neuroscience Institute, University of Sheffield, Sheffield S10 2HQ, UK

**Keywords:** Amyotrophic lateral sclerosis, Duchenne muscular dystrophy, Raman spectroscopy, biomarker, muscle

## Abstract

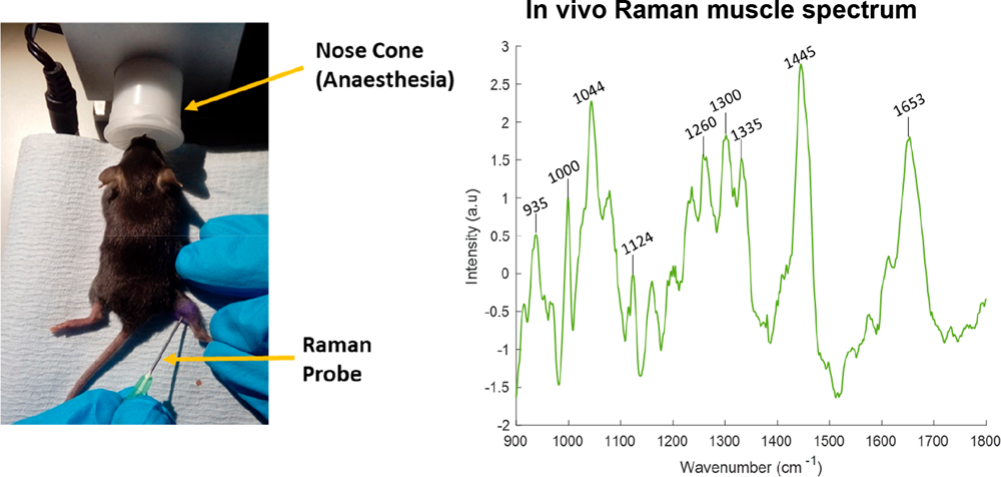

Neuromuscular diseases result in muscle weakness, disability, and, in many instances, death. Preclinical models form the bedrock of research into these disorders, and the development of *in vivo* and potentially translational biomarkers for the accurate identification of disease is crucial. Spontaneous Raman spectroscopy can provide a rapid, label-free, and highly specific molecular fingerprint of tissue, making it an attractive potential biomarker. In this study, we have developed and tested an *in vivo* intramuscular fiber optic Raman technique in two mouse models of devastating human neuromuscular diseases, amyotrophic lateral sclerosis, and Duchenne muscular dystrophy (SOD1^G93A^ and *mdx*, respectively). The method identified diseased and healthy muscle with high classification accuracies (area under the receiver operating characteristic curves (AUROC): 0.76–0.92). In addition, changes in diseased muscle over time were also identified (AUROCs 0.89–0.97). Key spectral changes related to proteins and the loss of α-helix protein structure. Importantly, *in vivo* recording did not cause functional motor impairment and only a limited, resolving tissue injury was seen on high-resolution magnetic resonance imaging. Lastly, we demonstrate that *ex vivo* muscle from human patients with these conditions produced similar spectra to those observed in mice. We conclude that spontaneous Raman spectroscopy of muscle shows promise as a translational research tool.

## Introduction

The development of biomarkers to identify and track pathology in disorders of nerves and muscles (termed collectively, neuromuscular disorders) is central to efforts aimed at improving the lives of patients with these disorders. For example, better determination of disease state would allow clinicians to intervene earlier in disease course, while also improving clinical trial design. Neuromuscular diseases cause considerable mortality and morbidity with annual per patient costs typically in excess of $50 000;^1^ as a result, these conditions are intensively researched in both preclinical and clinical settings. Two examples are amyotrophic lateral sclerosis (ALS) and Duchenne muscular dystrophy (DMD).

ALS is an incurable neurodegenerative disorder characterized by dysfunction and death of motor neurones in the brain and spinal cord, resulting in muscle weakness and death.^2^ Interestingly, there is evidence that muscle pathology can occur independently of neurone loss and starts early in disease,^3,4^ making muscle an attractive target for biomarker development. Preclinical models of the disease, usually mouse models, are crucial for understanding the pathobiology of the condition. Of those available, mice overexpressing the human mutant superoxide dismutase-1 (SOD1) gene are the best studied.^5^ However, it is now recognized that a lack of sensitive biomarkers of disease has hampered ALS preclinical studies.^6^*In vivo* biomarkers presently in use include time-consuming gait analysis systems and phenotype scores,^7^ expensive imaging tools, such as magnetic resonance imaging (MRI),^8^ and challenging electrophysiological measures.^9^

While ALS is typically encountered in the second half of life, DMD is the most common, fatal muscle wasting disease of childhood with a birth prevalence of up to 19.5 per 100 000 live births.^10^ It is an X-chromosome linked recessive disease caused by mutations (most commonly deletions) in the dystrophin gene. The ensuing absence/reduction in the cytoskeletal protein dystrophin results in muscle damage caused by repeated contractions. The *mdx* mouse is the most studied model of DMD.^11^ The *mdx* phenotype is relatively mild, and as a result, there are few *in vivo* methods for studying disease onset and progression. Techniques such as wire hanging and grip strength struggle to identify the mild symptoms and are confounded by animal weight and behavior;^12^ furthermore, some papers report using groups of up to 100 mice.^13^ As a result, muscle force studies and histological studies are done after sacrificing the animals in order to definitively study muscle pathology. Sensitive *in vivo* detection of the disease state therefore remains an area of unmet need.

Spontaneous Raman spectroscopy uses laser light of a single frequency to stimulate the vibrational modes of molecules within a sample. When assessing tissue samples, the resulting Raman spectrum provides a “biochemical fingerprint”.^14^ Biomedical applications of the technology are achieving sensitive, real-time tissue analysis across different tissues in cancer.^15−19^ Pertinent to the present study, a recent report used stimulated Raman scattering microscopy *in vivo* to study nerve pathology in several mouse models of ALS.^20^ In addition, spontaneous Raman has been used to study the spinal cord in the SOD1^G93A^ model^21^ and to examine fly models of human muscle diseases (myopathies).^22^ While these studies require either technically challenging *in vivo* methods or rely on *ex vivo* analysis, they demonstrate the potential of the technique for the study of neuromuscular disorders. Indeed, there are few *in vivo* techniques capable of providing the molecular information obtained through Raman spectroscopy.

Our aim in this study was to develop and test a methodology for identifying muscle pathology *invivo* in different mouse models of fatal neuromuscular diseases. Detecting the weak Raman signal within living tissue poses technical challenges relating to miniaturization, targeting the light to the organ of interest, photoluminescence, and spectral artifacts from probe components (e.g., silica). We have overcome these challenges and developed a simple, minimally invasive fiber-optic-based method for the label-free study of living muscle in preclinical models of devastating human diseases. Together with data from *ex vivo* human patient samples, the results highlight the growing potential of Raman spectroscopy as a translational biomedical research tool.

## Results and Discussion

**Figure 1:**
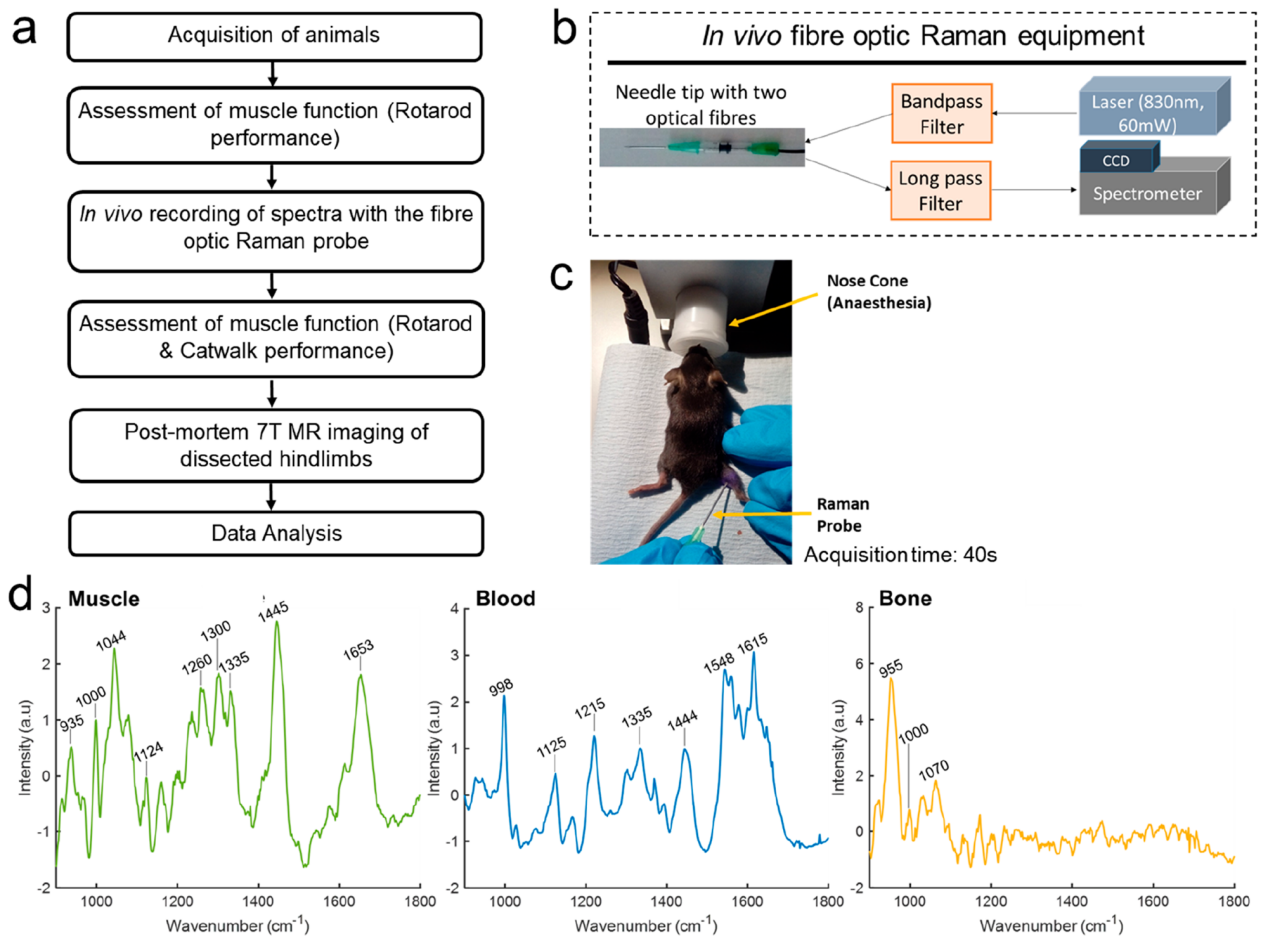
Intramuscular, *in vivo*, fiber optic Raman spectroscopy assessment. (a) A flowchart of the experiments. (b) A schematic of the fiber optic Raman system. (c) A mouse undergoing the procedure; the laser light can be appreciated within the hindleg as the camera used does not filter out the light. (d) Raman spectra obtained from muscle, blood, and bone. See Supporting Information Table 1 for tentative peak assignments and references.

**Figure 2:**
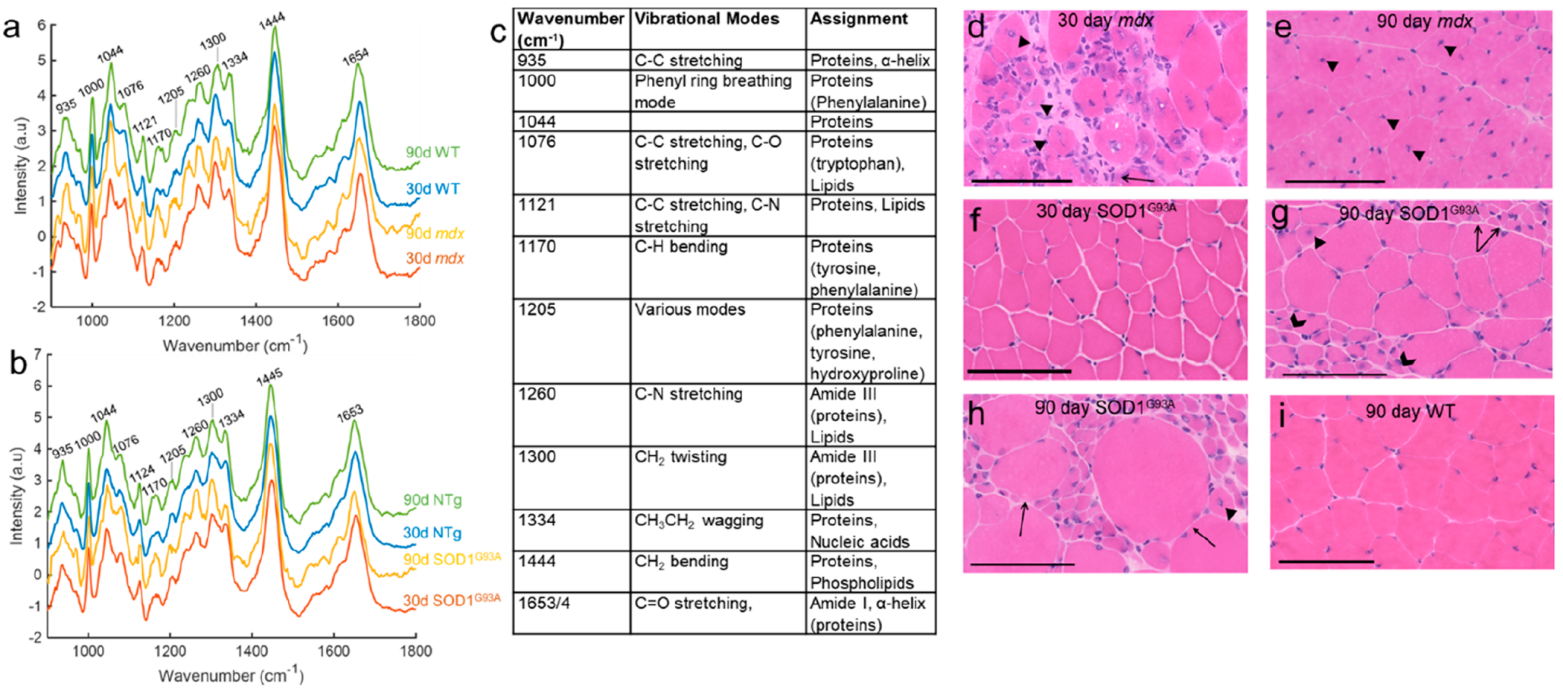
Intramuscular, *in vivo* Raman spectra and muscle histology. (a, b) Average spectra are shown for *mdx* and SOD1^G93A^, together with the relevant WT/NTg control for 30 and 90 days of age. Prominent peaks across the different groups are labeled (a and b). (c) Prominent Raman peaks in baseline subtracted spectra (present also in non-baseline subtracted spectra; Supporting Information Figure 2) and tentative peak assignments. See Supporting Information Table 1 for peak references. (d) Histological assessment at 30 days in *mdx* reveals necrotic fibers with inflammatory cells (arrow) and evidence of early regeneration (small myofibers with central nuclei, arrow heads). (e) The 90 day *mdx* muscle is characterized by regenerated myofibers, larger cells with central nuclei (arrow heads). (f) The 30 day SOD1^G93A^ muscle appears normal. The myofibers have peripheral nuclei and a regular shape. (g, h) The 90 day SOD1^G93A^ muscle displays evidence of denervation in the form of grouped atrophy (g, double arrow), small angular fibers (g, chevrons), and hypertrophic fibers (h, arrows) and centrally placed nuclei (g, h, arrowhead). (i) Normal myofibers from a 90 day WT mouse. Magnification for all images = x40, all scale bars = 100 μm. WT = wild type, NTg = nontransgenic.

Using the SOD1^G93A^ model of ALS and *mdx* model of DMD, we were able to record spectra from living muscles, which were clearly different from those of neighboring tissues such as blood and bone (Figure 1, Supporting Information Figure 1). Tentative peak assignments for all tissues can be found in Supporting Information Table 1. Mean muscle spectra, both with (Figures 1 and 2) and without (Supporting Information Figure 2) baseline subtraction, comprised peaks relating to the key constituents of muscle such as myosin, tropomyosin, and actin (Supporting Information Table 2). As expected, these prominent peaks were common to muscle from both murine models. Muscle histology demonstrated both the underlying similarity of the muscle and the pathological features of each model (Figure 2).

In order to test the ability of the *in vivo* intramuscular method to detect muscle disease and visualize more subtle differences in the spectra, we employed a range of multivariate techniques. Results for two-group PCA-LDA are shown in the main text (Table 1); data from PCA-QDA and PLS-DA are presented in Supporting Information Tables 3–5.

**Table 1:** Classification of Different Neuromuscular Disease Models^a^

	sensitivity (±SD)	specificity (±SD)	AUROC (±SD)
30 day SOD1^G93A^/ vs WT			
90 day SOD1^G93A^/ WT	82.5 (±2.0)	78.0 (±3.1)	0.86 (±0.01)
30 day/90 day SOD1^G93A^	85.5 (±3.8)	86.6 (±2.5)	0.92 (±0.01)
30 day female *mdx*/ WT	71.3 (±3.1)	65.7 (±2.6)	0.76 (±0.02)
90 day female *mdx*/ WT	91.6 (±2.2)	76.4 (±3.0)	0.91 (±0.01)
30 day/90 day *mdx* (female)	95.6 (±1.8)	71.6 (±3.4)	0.92 (±0.01)
30 day female *mdx*/ SOD1^G93A^	89.9 (±1.8)	97.1 (±2.0)	0.97 (±0.01)
90 day female *mdx*/ SOD1^G93A^	93.5 (±2.6)	73.3 (±1.7)	0.89 (± 0.02)

a The different comparisons are shown along with the classification performance parameters from PCA-LDA. In the 30 day SOD1 vs WT analysis, there were no significant PCs identified; thus, no model was generated.

**Figure 3:**
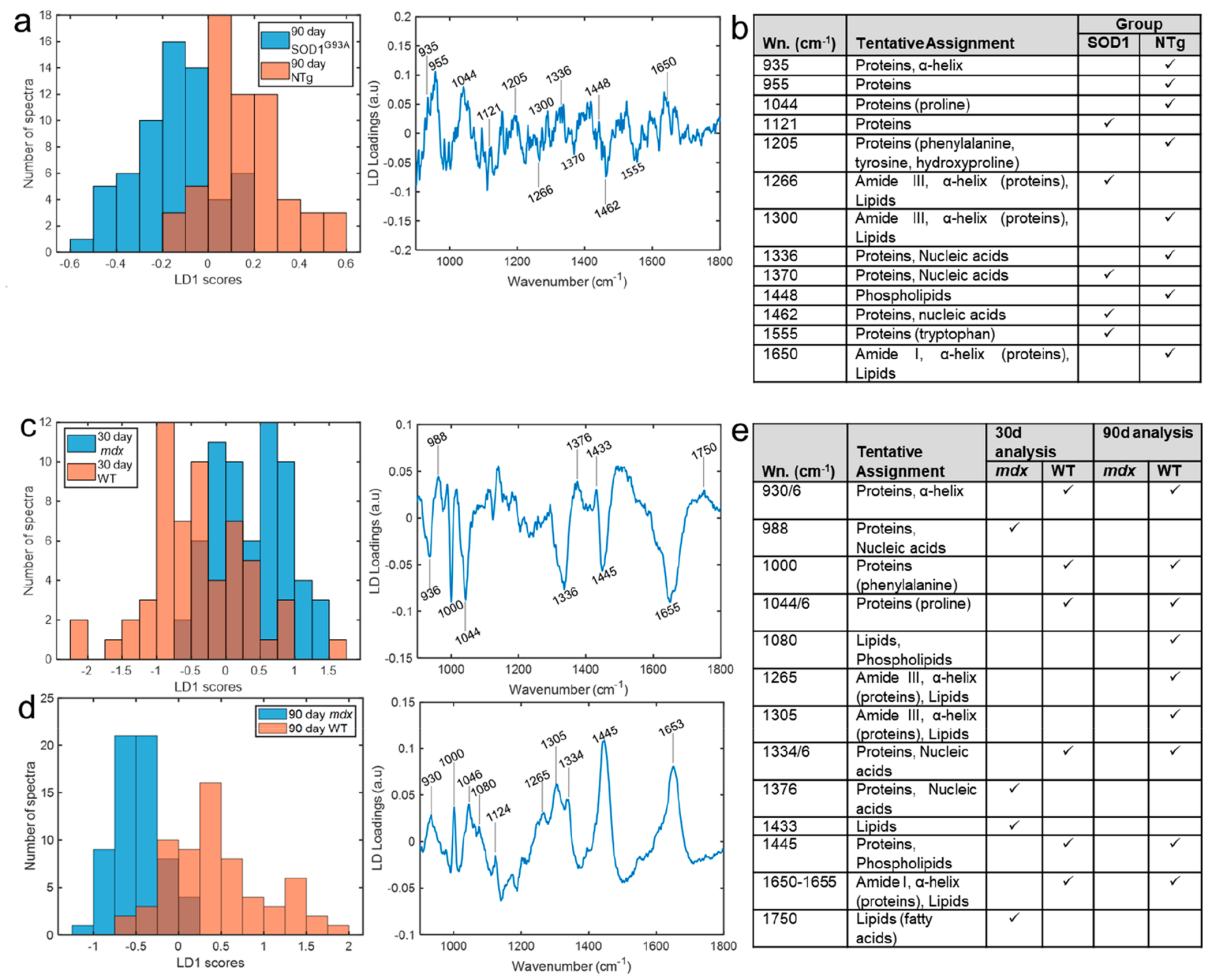
Linear discriminant function histograms and loadings plots. (a) Linear discriminant function (LDF) histogram and associated loadings plot for the comparison between 90 day SOD1^G93A^ and NTg mice. (b) Tentative peak assignments for the 90 day SOD1^G93A^ vs NTg comparison. (c) LDF histogram and associated loadings plot for the comparison between 30-day *mdx* and WT mice. (d) LDF histogram and associated loadings plot for the comparison between 90 day *mdx* and WT mice. (e) Tentative peak assignments for the *mdx* vs WT comparisons. Peak references can be found in Supporting Information Table 1. Wn = wavenumber.

In the SOD1^G93A^ model, the 30 day time-point is prior to disease onset; by 90 days, the mice have established disease.^7,23,24^ This was apparent in the classification performance. At 30 days, discrimination between transgenic (Tg) and non-transgenic (NTg) mice was not possible; however, at 90 days (Tg vs NTg) and between 30 and 90 day Tg mice, high classification performances (AUROC ≥ 0.86) were observed (Table 1, Supporting Information Figure 3). Examination of muscle histology is in keeping with these results, with no pathology evidence at 30 days but marked evidence of denervation at 90 days (Figure 2). The linear discriminant (LD) score plots for these comparisons are shown as histograms, together with the associated loading plots and tentative peak assignments (Figure 3 and Supporting Information Figure 4). Average and difference spectra demonstrated reduced concentrations of phenylalanine (ring breathing mode, 1000 cm^–1^), proteins (935, 1044, and 1444 cm^–1^), and the C=O stretching of amide I (1654 cm^–1^) in disease states (Supporting Information Figures 2 and 5).

The *mdx* mouse undergoes an acute onset of disease at approximately 30 days of age characterized by inflammation and necrosis, followed by highly effective regeneration^25^ (Figure 2). We observed a high classification performance at both 30 and 90 days of age (Table 1, Supporting Information Figure 3). Comparison of the *mdx* and SOD1^G93A^ models at both 30 and 90 days of age demonstrated accurate identification (AUROC > 0.89) of each disease model (Table 1).

Examination of the LD loadings for the different analyses revealed a key spectral fingerprint for muscle health across both mouse models, comprising similar peaks to those identified in the difference spectra (Figure 3, Supporting Information Figures 2 and 4). Tentative assignments are given in Figure 3. When comparing SOD1^G93A^ and *mdx* with each other at 30 days, a similar picture emerges, with key muscle health peaks attributed to the presymptomatic SOD1^G93A^ muscle (Supporting Information Figures 2 and 6). At 90 days, when muscle pathology in both models is established, *mdx* mice manifest greater losses in protein-related peaks, and a more complex picture emerges in the multivariate analysis, with shifts in proteins and lipids across both groups seen (Supporting Information Figure 6). Lastly, four group models, comprising 30- and 90-day-old disease-only mice (i.e., SOD1^G93A^ and *mdx* mice), also demonstrated a high classification performance (Supporting Information Figures 7 and 8).

**Figure 4:**
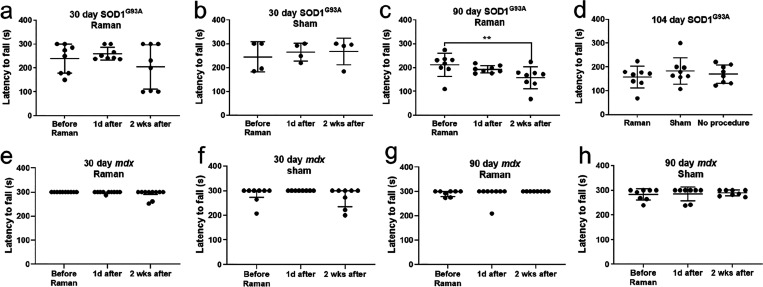
Post-*in vivo* Raman spectroscopy motor performance. Rotarod performance for 30 day SOD1^G93A^ mice for both Raman (a) and sham (b) experiments. At 90 days of age, a significant decline in performance was seen two weeks postprocedure (c). However, there was no difference in rotarod performance at this final age (104 days) between mice undergoing Raman, sham, and no procedure (d), suggesting a disease-related decline. No change in rotarod performance was seen at either 30 days (e, f) or 90 days (g, h) in the *mdx* mice. ** = *P* < 0.01. d = day; wks = weeks.

If *invivo* Raman spectroscopy of muscle is to be used in studies with models such as SOD1^G93A^ and *mdx*, then it is important that motor function is not compromised by the procedure. Motor function testing utilizing the rotarod demonstrated no significant change in performance at 1 day after either a Raman or a sham procedure, for any of the disease groups tested (Figure 4; Supporting Information Figure 9). In the subset of mice undergoing a further recording at 2 weeks postprocedure, no change in performance was seen in *mdx* (Figure 4). A significant decline in performance was seen for the 90 day SOD1^G93A^ group (Figure 4c). Our previous work has demonstrated a prominent decline in disease-related motor performance between 90 and 114 days,^7^ and so we also tested a cohort of mice that did not undergo either the Raman or sham procedure (Figure 4d). No significant difference in rotarod performance was seen across Raman, sham, and no procedure groups, indicating a disease-related performance decline. In addition, a comprehensive assessment of gait using the catwalk gait analysis system did not demonstrate any systematic effect (Supporting Information Figure 10).

**Figure 5:**
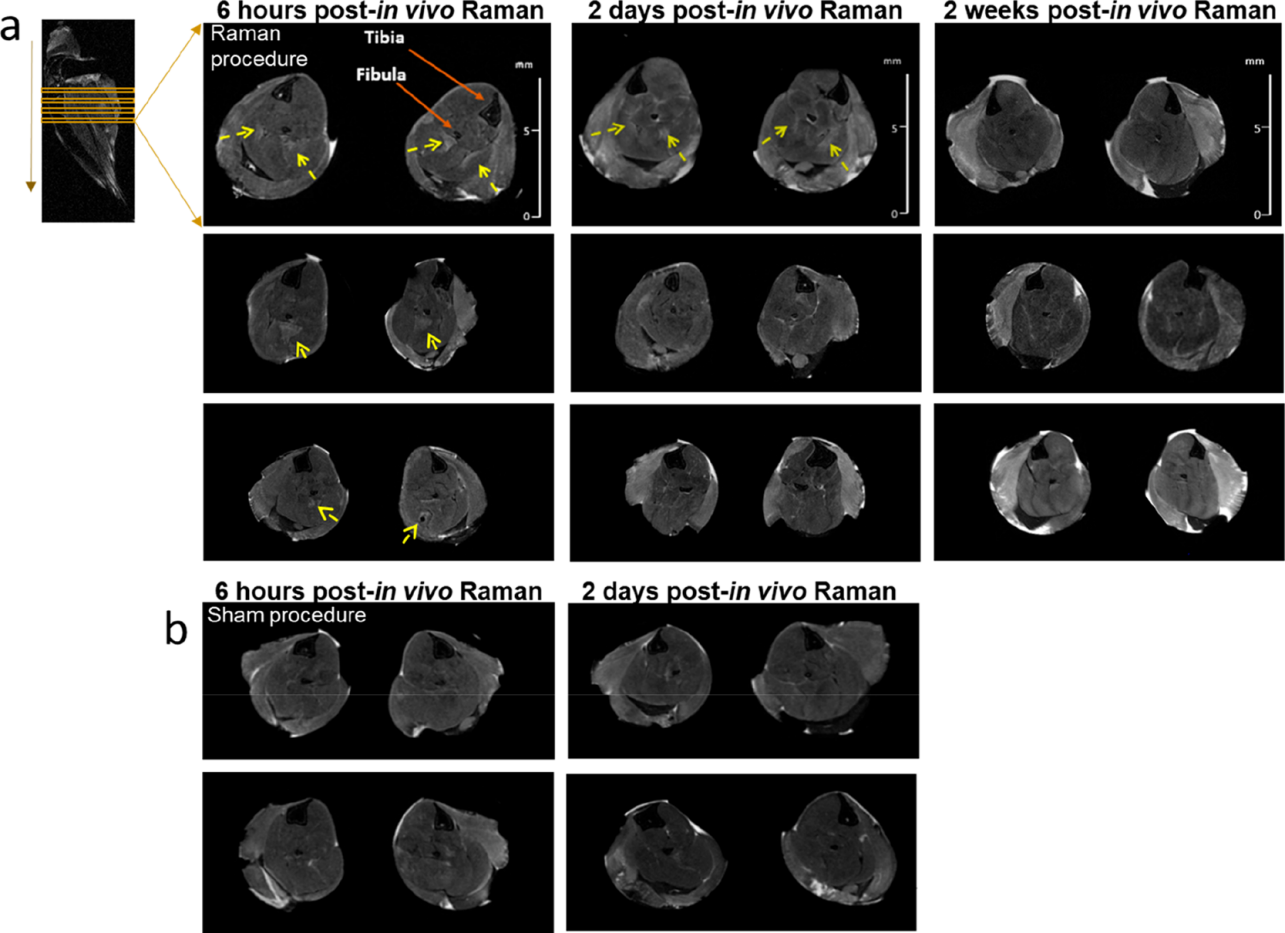
7T MRI evaluation of muscle that has undergone *in vivo* Raman spectroscopy. (a) Axial MRI (*ex vivo*) from NTg (healthy) mice. Muscles were studied at three different time points post-Raman measurement. Each panel represents a different mouse. The yellow arrows denote high T2 signal which may be due to postprocedure edema. (b) Axial MRI (*ex vivo*) from two time points following a sham procedure. Each panel represents a different mouse.

Assessment for subclinical muscle injury was performed using high-resolution MRI. WT mice were used to avoid pathological changes relating to disease complicating image interpretation. In mice undergoing MRI at 6 h post-Raman (*n* = 3), we observed subtle T2 hyperintensities in the gastrocnemius muscles around the site of needle placement (Figure 5). In one mouse, similar changes were apparent at 2 days; no changes were seen in any mice at 2 weeks postprocedure. No signal changes were apparent in mice undergoing the sham procedure (Figure 5). These results may suggest that laser exposure induces a degree of thermal injury, although we were only able to scan a small number of mice in both the Raman and sham groups.

**Figure 6:**
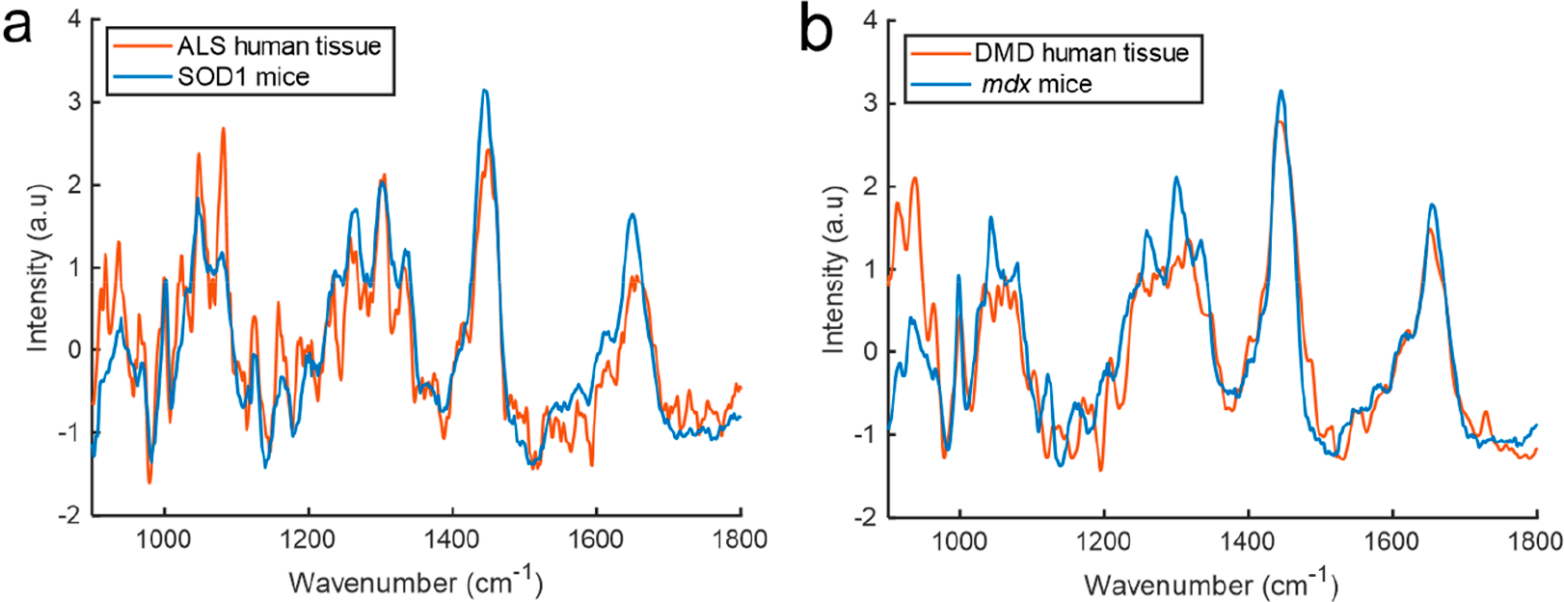
Human muscle spectra (*ex vivo*) and corresponding *in vivo* mouse model spectra. (a) Average *ex vivo* muscle spectra from patients with MND (orange) and *in vivo* spectra from 90 day-old SOD1^G93A^ mice (blue). (b) Average *ex vivo* muscle spectra from patients with DMD (orange) and *in vivo* spectra from 90 day-old *mdx* mice (blue).

Finally, to explore the translation potential of fiber optic Raman spectroscopy we undertook *ex vivo* recordings from human muscle samples obtained from patients with ALS (*n* = 3) and DMD (*n* = 3) (Figure 6). The *ex vivo* human spectra appeared similar to the *in vivo* mouse spectra. The limited availability of muscle tissue from children with muscle diseases means that a robust assessment of the classification performance between ALS and DMD human samples is not possible with these data. However, differences in the spectra were apparent, even without chemometric analysis (see also Supporting Information Figure 11).

Our results demonstrate that *in vivo* fiber optic Raman spectroscopy can identify muscle pathology in murine models of human neuromuscular disease. The simplicity of Raman spectroscopy offers potential as a translational biomarker in both preclinical and clinical studies.

In analyses based on spectral differences (both simple subtraction and multivariate statistics), a key fingerprint for muscle health emerged. Peaks associated with phenylalanine (1000 cm^–1^) and α-helical protein content (e.g., 935, 1300-5, 1654 cm^–1^) were seen to reduce in disease states, including with progression of the disease. These protein changes may represent the relative loss of the long α-helical structures within muscle proteins,^26^ as well as membrane phospholipids and proline, which act to enhance protein synthesis in muscle.^27^ When comparing fly models of different human muscle diseases, Gautam et al. also observed a reduction in α-helix content in the disease models.^22^ Of note, transcriptome studies in SOD1 mouse models of both ALS and DMD indicate upregulation of genes associated with protein degradation.^28−30^ As α-helices are the most abundant structures in proteins, our results would be in keeping protein breakdown. In addition, oxidative stress, known to occur in the muscles of both mouse models,^31,32^ has been shown to cause similar structural changes in different tissues/cells.^33−35^

Our methodology did not appear to result in any functional impairment using two standard methods of monitoring skeletal muscle function (rotarod and catwalk). In the catwalk analyses, a small number of postprocedure changes were observed, but these were not consistent. While catwalk is commonly used in studies concerning muscle injury and neuromuscular function, there are concerns regarding the reliability of the tests.^36^ Some authors suggest only using a certain set of parameters, none of which manifested changes in our analyses. Similarly, we did not observe changes in gait parameters documented by others after muscle injury.^37,38^ Overall, the lack of effect on motor function suggests that our approach can be incorporated into preclinical studies.

MRI studies demonstrated a transient change in T2 muscle signal postprocedure, which likely reflects edema cause by a resolving inflammatory response. In murine models of muscle injury and human subjects after intramuscular injection, similar time-limited changes in T2 signal intensity have been reported.^39−41^ We did attempt to undertake *in vivo* imaging with a view to complete serial scans in the same mouse, which would have allowed more mice to be imaged; however, long scan times failed to produce the desired resolution (data not shown).

Our approach has several strengths that make it appealing for use in future preclinical studies. The procedure can be performed under a standard anesthetic protocol and is quick to perform. The collection of Raman spectra took no more than 3 min; this contrasts to recording times of around 1 h required for *in vivo* MRI.^8^ Unlike new *in vivo* confocal imaging approaches,^42^ our Raman spectroscopy method requires no tissue preparation or prelabeling.

In the present study, we made several compromises in the *in vivo* recording protocol to reduce the potential for tissue damage. Probe diameter was limited to 0.5 mm to reduce the trauma of the insertion. A larger probe with a higher etendue for light collection may facilitate a better signal-to-noise ratio, as might longer acquisition times and/or increased laser power. However, the latter two would increase thermal energy exposure and possibly exacerbate any tissue injury. In this regard it is encouraging that *in vivo* human studies across a range of tissues and using a variety of laser powers/probe sizes have not reported any significant tissue damage.^43,19,44^ The fiber optic probe, operating at 60 mW laser power, has a maximum power density of 7.6 Wmm^–2^ at the distal tip, which is orders of magnitude below that typically used in conventional microscope-based systems. Furthermore, we only observed a transient tissue damage known to occur with procedures involving needle penetration of muscle.^40,45^ The relative size of the needle to the muscle being studied is also much larger in mice than would be the case in most human muscles. Thus, *in vivo* human recordings should be technically and practically feasible, although as with any new application of biophotonics, widespread uptake of the technique is likely to take time to realize.

In summary, our results demonstrate the use of minimally invasive fiber-optic spontaneous Raman spectroscopy for the *in vivo* assessment of muscle in preclinical models of neuromuscular diseases. The technique accurately distinguished between different types and stages of muscle disease. No impairment of motor function was seen, making it amenable for incorporation into preclinical drug studies. Analysis of *ex vivo* human muscle specimens demonstrated the translational potential of the technique. Spontaneous Raman spectroscopy of muscle is a promising biomarker for devastating human neuromuscular diseases.

## Methods

Details on the animal models used, human muscle samples, MRI methodology, histology, and motor function assessments can be found in the Supporting Information. Further details on data analysis can also be found in the Supporting Information.

### *In Vivo* Fiber Optic Raman Spectroscopy

Mice were anaesthetised using 2% isoflurane and positioned on a heat pad to maintain body temperature. The hindlimbs were shaved, and the fiber optic needle probe was inserted into the medial and lateral heads of both gastrocnemius muscles (Figure 1). The total time under anesthesia was 4 min.

The novel miniature (0.5 mm) fiber optic Raman probe was housed inside a standard 21 guage hypodermic needle. The design was based on that presented by Day and Stone^46^ and is shown schematically in Figure 1. A 830 nm semiconductor laser (Innovative Photonics Solutions) was used to provide a superior signal/noise ratio due to lower background fluorescence than shorter wavelength excitation.^19,47^ In line filters were placed approximately 15 cm from the probe tip to reduce the effect of Raman and fluorescence generated in the delivery fibers. These comprised a bandpass filter on the excitation delivery fiber and a long-pass filter on the collection fiber (Semrock Inc.). All fibers were low OH, with a 105 μm silica core of NA 0.22. The probe was optically matched to the spectrometer (Raman Explorer Spectrograph, Headwall Photonics, Inc. and iDus 420BR-DD CCD camera, Andor Technology, Ltd.) for optimum efficiency. A robust Raman signal of interest was recorded through a 40 s exposure consisting of 10 × 4 s epochs which were averaged. Beam divergence, with a 60 mW laser power at the probe tip and a near-infrared laser, reduces the potential for tissue damage through a lower energy density.^14^ Spectra from PTFE were acquired for wavenumber calibration. Following PTFE offset correction, an air background signal was recorded to check consistency across measurements. Spectra were acquired over a 2 year period with multiple groups of mice (i.e., both SOD1^G93A^/*mdx* and different ages) tested at each sitting to avoid any unknown factors confounding the results of a single group. All comparisons between disease and WT/NTg used *n* = 16 mice in each group. For assessment of muscle injury, a sham procedure was also used in which mice underwent the same procedure as above, with the exception that the laser was not switched on.

### Data Analysis

Over 6000 spectra were available for analysis, which was done using MATLAB (MATLAB R2019b The MathWorks, Inc., Natick, MA). Raw spectra were interpolated between 900 and 1800 cm^–1^, normalized using standard normal variate normalization (SNV)^48^ and mean-centered. Spectra were windowed between 900 and 1800 cm^–1^, as outside this region, the spectra were dominated by peaks and background related to the silica in the optical fibers (before 900 cm^–1^) or consisted of uninformative noise (after 1800 cm^–1^). Human tissue spectra (but not mouse spectra) were smoothed using a fifth order Savitzky–Golay filter^49^ (window width = 15 data points).

Principal component analysis-fed linear discriminant analysis (PCA-LDA), principal component analysis-fed quadratic discriminant analysis (PCA-QDA), and partial least-squares discriminant analysis (PLS-DA) classification models were generated for all the data sets. The first 10 principal components (PCs) were interrogated for significant between group differences using student’s *t* test or analysis of variance (ANOVA), followed by false discovery rate (fdr) correction (*Q* = 0.05). Those PCs demonstrating significant differences between the groups under assessment were used as inputs to LDA. Linear discriminant functions (LDFs) using PCs identified as statistically significant were plotted in order to illustrate the important peaks for spectral classification. For PLS-DA (Supporting Information), selection of the optimal number of latent variables was done by increasing their number until prediction accuracy no longer increased. The classification performance of the different models was validated using repeated leave-some-mice-out-cross-validation (RLSMOCV). The analysis was repeated 100 times using randomly selected combinations of left-out-mice. Area under the receiver operating characteristic curve (AUROC) was calculated. These curves plot the true positive rate (sensitivity) against the false positive rate (1-sensitivity) and illustrate the ability of a classifier to separate classes. As performance across the different classification algorithms was similar, PCA-LDA results are shown in the main text using linear discriminant loadings plots.^14^ These combine the information from relevant PCs, thus providing a more comprehensive exploration of the spectral features underlying class differentiation than single PC or latent variable loadings plots.

## Supporting Information Available

Additional methods; tabulation of tentative peak assignments and references; prominent Raman peaks; two-group classification for SOD1^G93A^ mice; two-group classification for *mdx* mice; two-group classification between SOD1^G93A^ and *mdx* mice; PC1 scores and loadings plots; average and difference spectra; ROC curves; linear discriminant function histograms and loadings plots; phenylalanine and α-helical protein content in disease comparisons; linear discriminant function histograms and loadings plots; supervised four group classification model results; unsupervised four group hierarchical clustering; additional rotarod data following Raman or sham procedures; catwalk gait analysis following the Raman procedure; and human ALS and DMD Raman spectra and tentative peak assignments. (PDF)

## Abbreviations

ALS: amyotrophic lateral sclerosis
AUROC: area under the receiver operating characteristic curve
DMD: Duchenne muscular dystrophy
FDR: false discovery rate
LD: linear discriminant
LDF: linear discriminant function
MRI: magnetic resonance imaging
NTg: nontransgenic
PC: principal component
PCA: principal component analysis
PCA-LDA: principal component analysis fed linear discriminant analysis
PCA-QDA: principal component analysis fed quadratic discriminant analysis
PLS-DA: partial least-squares-discriminant analysis
SNV: standard normal variate
SOD1: superoxide dismutase-1
Tg: transgenic
Wn: wavenumber
