# Supplementary material for: In Vivo Fiber Optic Raman Spectroscopy of Muscle in Preclinical Models of Amyotrophic Lateral Sclerosis and Duchenne Muscular Dystrophy
Source: ACS Chem Neurosci. 2021 May 5;12(10):1768–76. doi: 10.1021/acschemneuro.0c00794 (PMC8154326; doi:10.1021/acschemneuro.0c00794)
Supplement: Supplementary file 1 — cn0c00794_si_001.pdf [file cn0c00794_si_001.pdf]

Supporting information

In vivo Fibre Optic Raman spectroscopy of Muscle in Preclinical Models of Amyotrophic Lateral Sclerosis and Duchenne Muscular Dystrophy

Maria Plesia[a], Oliver A. Stevens[b], Gavin R. Lloyd[c,d], Catherine A. Kendall[d], Ian Coldicott[a], Aneurin J. Kennerley[e], Gaynor Miller[f], Pamela J. Shaw[a,g], Richard J. Mead[a,g], John C. C. Day[b], James J.P. Alix[a,g*]

[a]Sheffield Institute for Translational Neuroscience, University of Sheffield

[b]Interface Analysis Centre, School of Physics, University of Bristol

[c]Phenome Centre Birmingham, University of Birmingham

[d]Biophotonics Research Unit, Gloucestershire Hospitals NHS Foundation Trust.

[e]Department of Chemistry, University of York

[f]Department of Oncology and Metabolism, University of Sheffield

[g]Cross-Faculty Neuroscience Institute, University of Sheffield

***Correspondence**

Dr James J.P. Alix

Sheffield Institute for Translational Neuroscience, 385a Glossop Road, Sheffield, S10 1HQ, UK.

Email: j.alix@sheffield.ac.uk

Supplemental methods

Ex vivo mouse tissue and human muscle Raman spectroscopy

Ex vivo recordings of mouse blood and bone were performed. Blood was obtained immediately upon culling the mice via cardiac puncture and Raman spectra obtained using the same parameters described above. For bone assessment, associated muscles were dissected away from the tibial bone and the probe placed directly onto the shaft of the bone

Post-mortem human biceps and quadriceps muscle from patients with an established diagnosis of ALS was obtained from the Sheffield Brain Tissue Bank. These patients included one male aged 39 years and 2 female patients aged 58 and 76 years. Quadriceps muscle tissue from patients with a genetic diagnosis of Duchenne muscular dystrophy was obtained from the Oxford Brain Bank. The boys were aged 2, 4 and 10 years. The fibre optic probe was pressed against the muscle samples and 3-5 areas sampled, depending upon the size of the specimen. Spectra were obtained using the same parameters described above. Human tissue use was approved by an NHS Research Ethics committee (reference 16/YH/0261).

MRI

NTg (C57bl/6) mice were culled at 6 hours, two days, or two weeks following the in vivo Raman recordings (n=3 at each time point). Following sacrifice with terminal anaesthesia, the gastrocnemius muscle were dissected and imaged immediately using a 7 Tesla magnet (Bruker BioSpecAVANCE III, 310 mm bore, MRI system B/C 70/30), with a 12 channel RT-shim system (B-S30) and preinstalled 660mT/m imaging gradient set (BGA-12S). A 1H birdcage volume resonator (Bruker, 300 MHz, 1 kW max, outer diameter 114 mm/inner diameter 72 mm), placed at the isocentre of the magnet, was used for both radiofrequency (RF) transmission and reception. The hindlegs of each mouse were placed in a PFPE lubricant oil (Fomblin) filled eppendorf for scanning. Following field shimming, off-resonance correction and RF gain setting a tri-plane Fast low angle shot (FLASH) sequence (Repetition time (TR) = 100 ms, Echo time (TE) = 6 ms, Flip angle = 30uo, Number of averages = 1, Field of view (FOV) = 80mm*80mm, slice thickness = 2mm, Matrix = 128*128, spatial resolution = 0.625mm*0.625mm) was used to localize the muscle of interest. Subsequently, a fast rapid acquisition with refocused echo (RARE) sequence permitted low in plane resolution coronal visualisation of the hind limb muscles (TR = 2000 ms, RARE factor = 4; effective TE= 28 ms, averages=2, FOV=25 mm*25 mm, Slice thickness = 1 mm, Matrix = 256*256, 9 slices contiguous interleaved slice; scan time ~4 mins). These images were used to plan axial high resolution RARE images (TR = 5500 ms, RARE factor = 8; effective TE= 56 ms, averages=45, FOV=20 mm*20 mm, Slice thickness = 0.3mm mm, Matrix = 256*256, 39 slices contiguous interleaved slices; scan time ~2 hours), covering the entire region of interest. Fat suppression was used to prevent high intensity fat signal from obscuring the signal of interest. In addition, control images were also obtained at 6 hours and 2 days following the sham procedures (i.e. fibres placed into muscle, but laser not turned on, n=2 at each time point).

Motor function assessments

Motor function was tested using a standard rotarod assessment. Training was first performed on three consecutive days prior to performance recording. The first recorded test was undertaken one week before Raman/sham recording (the baseline assessment). Following the Raman procedure, rotarod test was performed by all mice at one day post-Raman. A subgroup of mice from each group were also tested at 2 weeks post-procedure. For the test, the rotarod (Ugo Basile 7650) was set to accelerate from 3 to 37 rpm in 270 seconds. Latency to fall was recorded in seconds for each mouse. Each mouse was allowed two runs and the best performance was used for the data analysis.

The catwalk gait analysis system version 7.1 was used to capture gait parameters. All mice were tested one week before our experimental procedure and one day after the recordings. Mice that were culled at two weeks post-recording were also tested prior to sacrifice. Animals were placed on the catwalk apparatus in complete darkness and gait patterns were recorded as they voluntarily traversed the glass plate. Only straight-line runs were acquired, a maximum of six runs were saved for each mouse and three were selected for analysis. Limb assignment was undertaken manually, and dedicated software then used for automated calculation of parameters.

Histology

Mice that were not utilised in MRI studies were sacrificed using isoflurane anaesthesia and cervical dislocation. Gastrocnemius muscles were dissected, rolled in optimal cutting temperature compound (OCT) and snap frozen in isopentane. Muscle cross-sections were obtained by cryosectioning at a thickness of 8 µm. Sections were stained with haematoxylin and eosin (H&E).

Data analysis

Power calculation:

The number of mice in each group (n=16; e.g. disease n=16 vs healthy n=16) was derived through a power calculation based on a preliminary analysis. This calculation was based the following assumptions (1):

- Two groups per comparison.
- 95% confidence interval (two sided; interval +/- 0.15).
- Prevalence is not applicable - set to be equal i.e. 1:1.
- Initial 70% sensitivity / specificity for all comparisons.
- The study will estimate Area under ROC curve (AUC) +/- interval.
- Four observations per mouse (i.e. two per hindlimb; clustered data).
- Assume moderate correlation between measurements from the same mouse = 0.5.

Previous work from our group with the $SOD1^{G93A}$ mice indicated that a 20% drop in performance can be detected at 80% power with n=14 ($\alpha = 0.05$, $\beta = 0.8$) for rotarod, and n=7 for catwalk gait analysis (2), thus alterations in these tests fall within the *n* number obtained from the Raman spectroscopy power calculation.

To calculate the percentage changes of phenylalanine and alpha helical protein content in disease states, the wavenumber with the largest intensity difference between the two groups was identified using the difference spectrum in the following four peak regions: 930-936, 998-1002, 1300-1305, 1650-1656 cm^{-1}. The initial value for that wavenumber (i.e. the value of either the healthy or younger age group) was then taken from the mean spectrum of this group. The percentage change was calculated: (largest difference/initial value)*100.

Four group modelling was undertaken using Orange Data Mining (3). For this analysis only $SOD1^{G93A}$ and *mdx* mice were included. Raw spectra were interpolated to integer wavenumber spacings between 900 and 1800 cm^{-1} and normalised using standard normal variate normalisation (SNV). As for other PCA-based analyses, the statistically significant principal components from the first 10 principal

components were used as inputs. k-nearest neighbour (k-NN) was performed using 3 neighbours, based on Euclidean distance (uniform distance weighting). Support vector machine (SVM) was performed using a linear Kernel. PCA-SVM and PCA-kNN were cross validated using leave-one-mouse-out cross validation. For hierarchical cluster analysis, principal component distances were calculated using the Euclidean distance, after which hierarchical clusters were obtained using the complete-linkage algorithm.

Raman Shift (cm^{-1})	Tentative Assignments	References
920	Proline, Glucose, Proteins	(4), (5), (6), (7), (8), (9)
932-940	Proline, Hydroxyproline, Proteins (α-helix)	(4), (5), (6), (8), (9), (10), (11), (12), (13), (14), (15), (16), (17), (18)
950-955	Hydroxyproline, Proline, Valine, Proteins, Hydroxyapatite, Carotenoids	(19), (20), (21), (22), (23), (24), (25)
980-988	Thymine, Uracil, Tryptophan, Proteins	(17), (18), (21)
1000-1006	Phenylalanine (phenyl ring breathing mode), Proteins	(4), (8), (11), (12), (15), (16), (17), (18),(21), (22), (26)
1014-1016	Tryptophan, Proteins	(7), (27)
1040-1045	Proline, Proteins	(6), (16), (18), (21), (22), (28)
1076-1080	Lipids, Phospholipids, Tryptophan, Nucleic acids	(4), (21), (24), (26), (29)
1100-1104	Uracil, Nucleic acids, Proteins, Lipids	(18), (19), (21), (22), (30)
1120-1128	Tryptophan, Valine, Proteins, Glucose, Lipids, Phospholipids	(4), (7), (8), (16), (17), (18), (22), (24), (31), (32), (33)
1165-1172	Tyrosine, Phenylalanine, Proteins	(6), (11), (12), (17), (20)
1200-1207	Tyrosine, Phenylalanine, Hydroxyproline, Proteins	(4), (6), (8), (18), (21), (22), (34)
1220-1227	Proteins, Amide III (β-sheet)	(4), (12), (20)
1260-1269	Proteins, Amide III (α-helix), Tyrosine, Tryptophan, Proline, Proteins, Fatty Acids, Phospholipids, Lipids	(4), (7), (15), (16), (22), (26), (29), (35), (36), (37), (38), (39)
1275-1310	Proteins, Amide III (α-helix)	(40)
1300-1305	Proteins, Amide III, Fatty acids, Phospholipids, Cholesterol, Lipids	(7), (11), (15), (16), (19), (20), (24), (28), (29), (33), (36), (40), (41), (42), (43), (44), (45)
1316-1320	Valine, Proline, Tryptophan Proteins, Phospholipids, Guanine	(6), (7), (21), (22), (26), (27)
1328-1336	Tryptophan, Valine, Proline, Proteins, Adenine, Guanine Nucleic acids	(4), (7), (11), (12), (15), (18), (21), (22), (28), (33), (46), (47), (48)
1370-1376	Saccharide, Adenine, Thymine, Guanine	(10), (21), (33), (49)
1420-1450	Cholesterol, Phospholipids Fatty acids, Triglycerides, Lipids, Proteins	(4), (8), (12), (15), (16), (17), (18), (19), (28), (29), (36), (42), (49), (50), (51), (52), (53), (54)
1460-1468	Proline, Tryptophan, Proteins, Deoxyribose, Cytosine, Guanine, Adenine, Nucleic acids	(7), (11), (17), (21), (22), (47)
1510-1516	Cytosine, Adenine, Guanine	(10), (47)

1548-1556	Tryptophan, Proteins	(4), (8), (16), (18), (21), (22)
1645-1656	Proteins, Amide I (α-helix), Phospholipids, Lipids	(4), (6), (11), (15), (16), (17), (18), (20), (26), (29)
1750	Phospholipids	(26)

Supplementary table 1.

Tentative peak assignments for peaks observed from Raman analysis of the SOD1^{G93A} and *mdx* mouse models (muscle, blood, and bone) and from human muscle samples.

**Wavenumber (+/-7 cm^{-1}) (cm^{-1})**	**Myosin** (16), (55)	**Tropomyosin** (17)	**Actin** (acquired from figure) (56)	**Type I Collagen** (6), (14), (11), (28), (48), (57)	**Muscle Fibre** (18)	**Skeletal Muscle** (15), (28), (32)
935	✓	✓		✓	✓	✓
1001	✓	✓	✓	✓	✓	✓
1044	✓				✓	
1076	✓		✓			
1124	✓	✓			✓	✓
1173	✓	✓			✓	✓
1205	✓	✓				
1264	✓			✓	✓	✓
1300	✓	✓		✓		✓
1335	✓	✓		✓	✓	✓
1445	✓	✓	✓	✓	✓	✓
1654	✓	✓	✓	✓	✓	✓

Supplementary table 2.

Prominent Raman peaks from *in vivo* intra-muscular fibre optic Raman spectroscopy compared with peaks of major muscle components and skeletal muscle.

	30 day SOD1^{G93A}/WT				90 day SOD1^{G93A}/WT				30 day SOD1^{G93A}/90 day SOD1^{G93A}			
Chemometric technique	Sens. (+/- s.d.)	Spec. (+/- s.d.)	Acc. (+/- s.d.)	AUROC (+/- s.d.)	Sens. (+/- s.d.)	Spec. (+/- s.d.)	Acc. (+/- s.d.)	AUROC (+/- s.d.)	Sens. (+/- s.d.)	Spec. (+/- s.d.)	Acc. (+/- s.d.)	AUROC (+/- s.d.)
PCA-LDA (shown in main document)	-	-	-	-	**82.5 (+/-2.0)**	**78.0 (+/-3.1)**	**79.3 (+/- 1.7)**	**0.86 (+/- 0.02)**	85.5 (+/- 3.8)	86.6 (+/-2.5)	85.9 (+/- 2.3)	0.92 (+/- 0.01)
PCA-QDA	-	-	-	-	78.8 (+/-2.8)	72.6 (+/-3.4)	75.8 (+/- 2.2)	0.81 (+/- 0.02)	83.9 (+/- 3.5)	86.6 (+/-2.7)	85.2 (+/- 2.2)	0.90 (+/- 0.02
PLS-DA	**43.3 (+/- 5.5)**	**43.8 (+/- 6.1)**	**43.2 (+/- 4.5)**	**0.42 (+/-0.05)**	80.0 (+/-2.7)	74.8 (+/-4.2)	77.4 (+/- 2.6)	0.85 (+/-0.02)	**94.3 (+/- 1.8)**	**96.9 (+/-2.0)**	**95.6 (+/- 1.5)**	**0.99 (+/- 0.005)**

Supplementary table 3. Classification performance for SOD1^{G93A} mice using different chemometric techniques.

The technique with the best classification performance is shown in bold.

	30 day *mdx*/WT				90 day *mdx*/WT				30 day *mdx*/90 day *mdx*			
Chemometric technique	Sens. (+/- s.d.)	Spec. (+/- s.d.)	Acc. (+/- s.d.)	AUROC (+/- s.d.)	Sens. (+/- s.d.)	Spec. (+/- s.d.)	Acc. (+/- s.d.)	AUROC (+/- s.d.)	Sens. (+/- s.d.)	Spec. (+/- s.d.)	Acc. (+/- s.d.)	AUROC (+/- s.d.)
PCA-LDA (shown in main document)	71.3 (+/-3.1)	65.7 (+/-2.6)	68.6 (+/- 2.0)	0.76 (+/- 0.02)	91.6 (+/-2.2)	76.4 (+/-3.0)	84.1 (+/- 1.7)	0.91 (+/- 0.01)	95.6 (+/-1.8)	71.6 (+/-3.4)	83.6 (+/- 2.0)	0.92 (+/- 0.01)
PCA-QDA	76.7 (+/-3.1)	63.1 (+/-1.8)	70.4 (+/- 1.9)	0.74 (+/- 0.02)	88.6 (+/-1.8)	78.4 (+/-2.0)	83.5 (+/- 1.2)	0.90 (+/- 0.01)	89.1 (+/-1.5)	77.6 (+/-2.5)	83.4 (+/- 1.5)	0.90 (+/- 0.01
PLS-DA	**80.5 (+/- 4.0)**	**71.6 (+/- 3.4)**	**76.3 (+/- 3.1)**	**0.85 (+/- 0.03)**	**94.2 (+/-2.3)**	**88.7 (+/-2.9)**	**91.3 (+/- 1.8)**	**0.96 (+/-0.01)**	**95.4 (+/- 1.6)**	**80.6 (+/-3.2)**	**88.0 (+/- 2.0)**	**0.96 (+/- 0.009)**

Supplementary table 4. Classification performance for *mdx* mice using different chemometric techniques.

The technique with the best classification performance is shown in bold.

	30 day *mdx*/90d $SOD1^{G93A}$				90 day *mdx*/90 day $SOD1^{G93A}$			
Chemometric technique	Sens. (+/- s.d.)	Spec. (+/- s.d.)	Acc. (+/- s.d.)	AUROC (+/- s.d.)	Sens. (+/- s.d.)	Spec. (+/- s.d.)	Acc. (+/- s.d.)	AUROC (+/- s.d.)
PCA-LDA (shown in main document)	89.9 (+/- 1.8)	97.1 (+/- 1.9)	93.4 (+/- 1.4)	0.97 (+/- 0.01)	93.5 (+/- 2.6)	73.3 (+/- 1.7)	83.5 (+/- 1.6)	0.89 (+/- 0.02)
PCA-QDA	89.3 (+/- 2.1)	95.3 (+/- 1.8)	92.2 (+/- 1.5)	0.96 (+/- 0.01)	87.4 (+/- 2.8)	78.5 (+/- 2.1)	83.0 (+/- 1.6)	0.89 (+/- 0.02)
PLS-DA	**93.6 (+/- 0.8)**	**97.3 (+/- 1.3)**	**95.3 (+/-1)**	**0.98 (+/- 0.004)**	**88.2 (+/- 3.8)**	**78.2 (+/- 4.8)**	**83.2 (+/- 3.0)**	**0.92 (+/- 0.02)**

Supplementary table 5. Classification performance for *mdx* vs. $SOD1^{G93A}$ mice using different chemometric techniques.

The technique with the best classification performance is shown in bold.

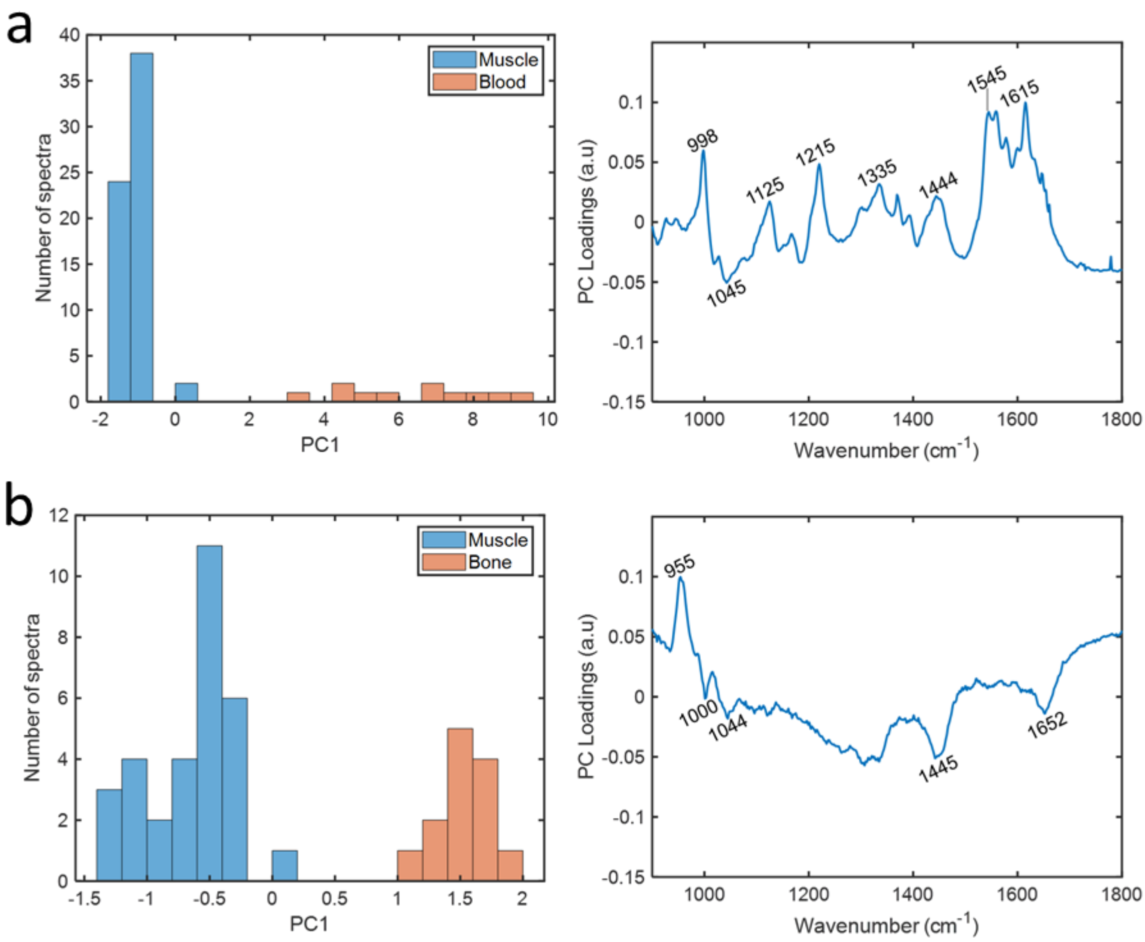

Supplementary figure 1. Comparison of muscle, blood and bone.

(a). PC1 score histogram and loading plot from comparison between muscle and blood.

(c). PC1 score histogram and loading plot from comparison between muscle and bone.

See supplemental table 1 for tentative peak assignments and references.

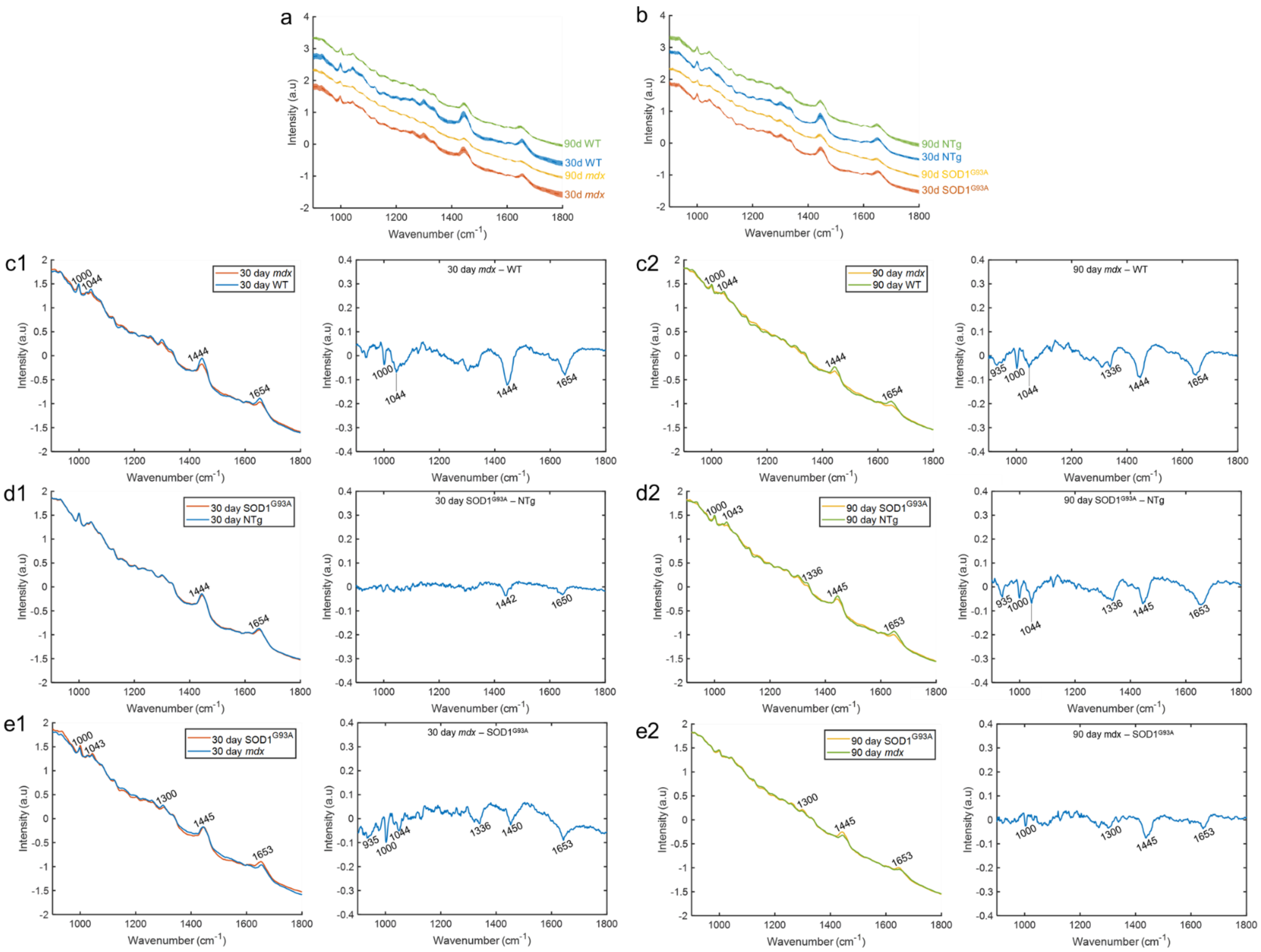

Supplementary figure 2. Average spectra and difference spectra.

(a & b). The mean spectra for mdx (a) mice and SOD1^{G93A} (b), plus relevant controls. The shaded areas represent (+/-) one standard deviation from the mean spectrum. The spectra have been offset for clarity.

(c1 & 2). Mean and difference spectra (without background subtraction) are shown for *mdx* and healthy control mice at both 30- and 90-days. Prominent peaks that differ between the respective groups are indicated.

(d1 & 2). Mean and difference spectra for SOD1^{G93A} and their respective controls at both 30 and 90 days.

(e1 &2). Mean and difference spectra between SOD1^{G93A} and *mdx* at both 30 and 90 days.

See supplemental table 1 for tentative peak assignments and references.

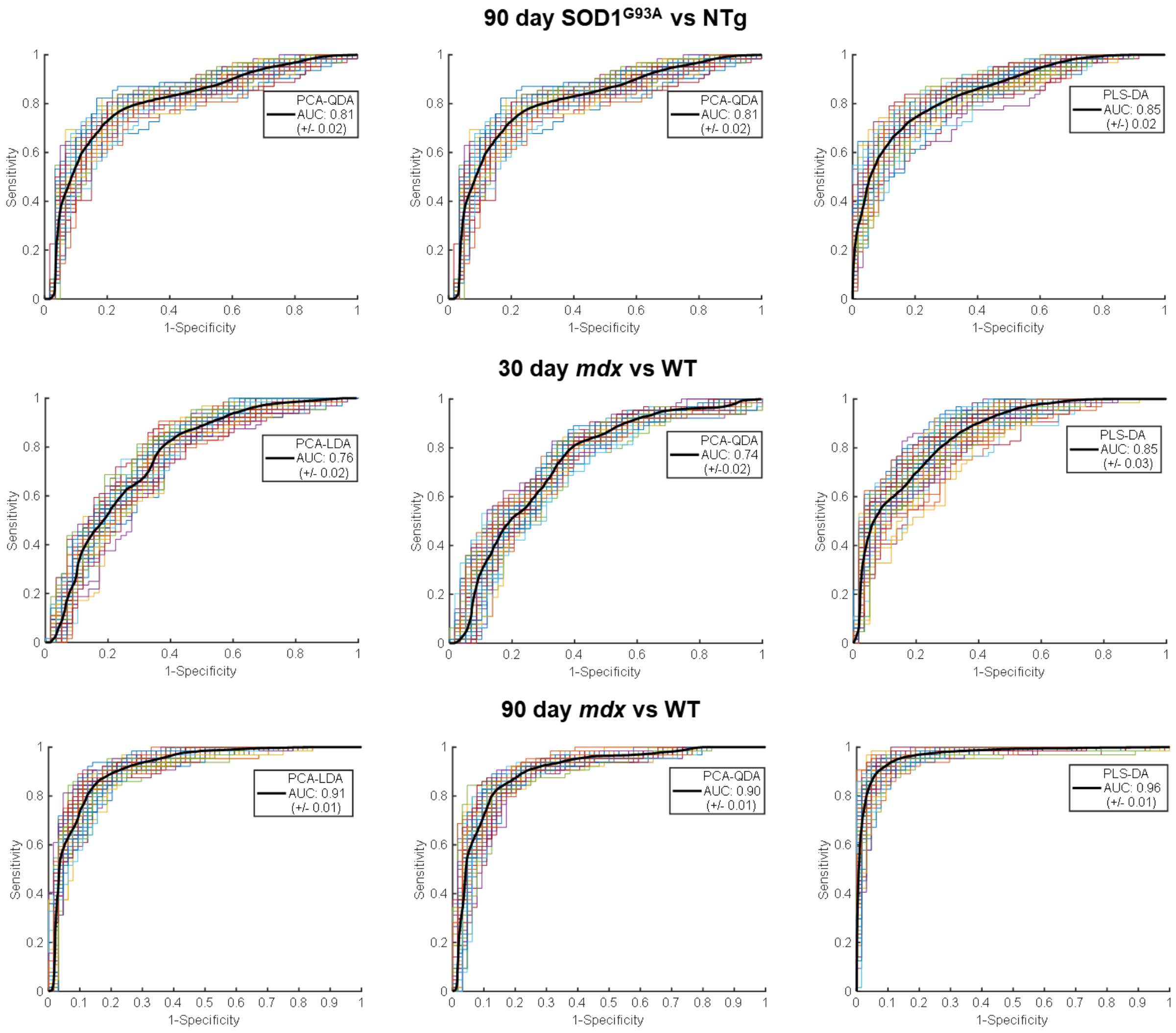

Supplementary figure 3. ROC curves for SOD1^{G93A} and *mdx* analyses.

The curves are generated from repeated leave-four-mice out cross validation. The cross validation is repeated 100 times with different combinations of mice are left out. The coloured lines represent the individual results, the black line the average performance.

a

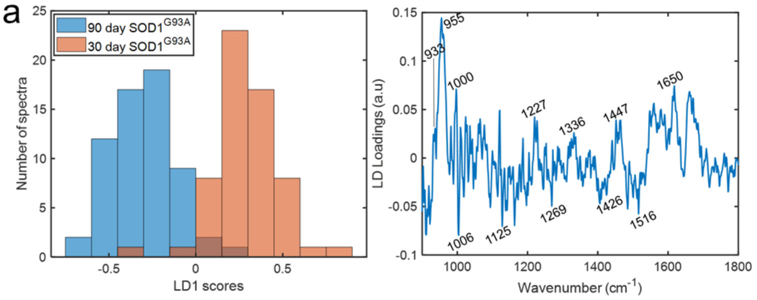

Wn. (cm-1)	Tentative Assignment	Group 30d	90d
935	Proteins, α-helix	✓	
955	Proteins	✓	
1000	Proteins (phenylalanine)	✓	
1006	Proteins		✓
1125	Proteins, Lipids		✓
1227	Amide III (Proteins)	✓	
1269	Amide III, α-helix (Proteins), Lipids		✓
1336	Proteins, Nucleic acids	✓	
1426	Deoxyribose		✓
1447	Phospholipids, Proteins	✓	
1516	Cytosine, Carotenoids		✓
1650	Amide I, α-helix (Proteins), Lipids	✓	

b

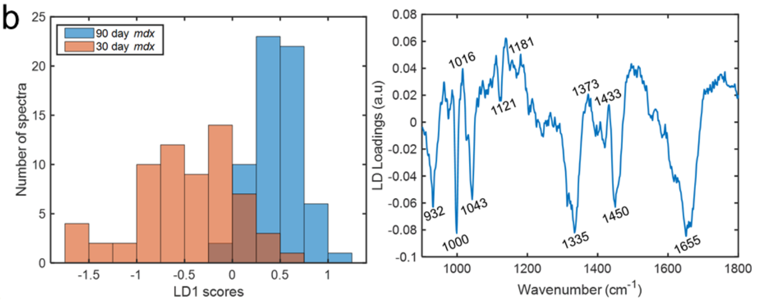

Wn. (cm-1)	Tentative Assignment	Group 30d	90d
932	Proteins, α-helix	✓	
1000	Proteins (phenylalanine)	✓	
1016	Tryptophan		✓
1043	Proteins (Proline)	✓	
1121	Proteins, Lipids		✓
1181	Cytosine, Guanine, Adenine		✓
1335	Proteins, Nucleic acids	✓	
1373	Ring breathing modes of the DNA/RNA bases		✓
1433	Lipids		✓
1450	Phospholipids, Proteins	✓	
1655	Amide I, α-helix (Proteins), Lipids	✓	

Supplementary figure 4. Linear discriminant function histograms and loadings plots for longitudinal disease comparisons.

(a). LD histogram, associated loadings plot and tentative peak assignments for the comparison between 30 and 90 day SOD1^{G93A} mice.

(b). LD histogram, associated loadings plot and tentative peak assignments for the comparison between 30 and 90 day *mdx* mice.

See supplemental table one for tentative peak assignments and references.

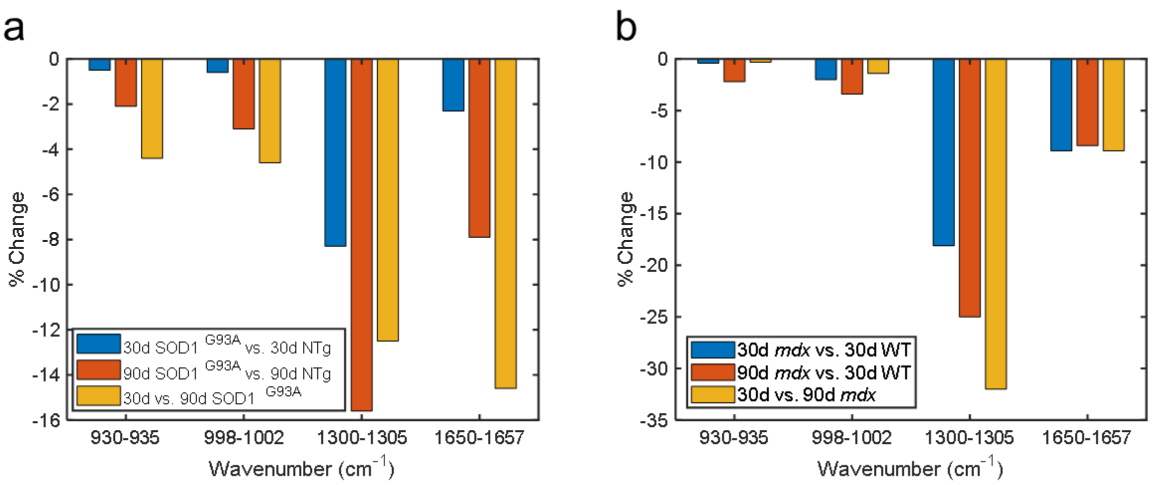

Supplemental figure 5. Phenylalanine and alpha helical protein content in disease comparisons.

(a). Percentage changes of phenylalanine and alpha helical protein peaks in SOD1 ^{G93A} comparisons.

(b). Percentage changes of phenylalanine and alpha helical protein peaks in *mdx* comparisons.

d = day.

a

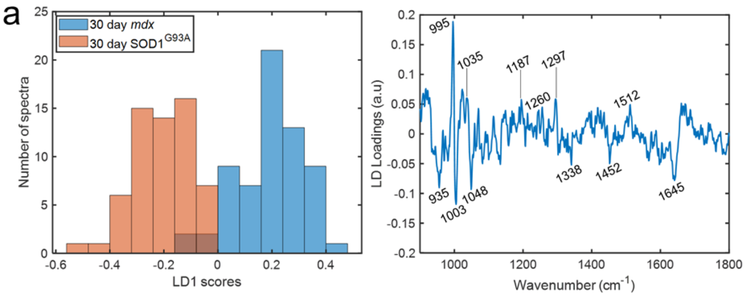

Wn. (cm-1)	Tentative Assignment	Group	
		mdx	SOD1
935	Proteins, α-helix		✓
995	Proline	✓	
1003	Proteins (phenylalanine)		✓
1035	Proteins (proline, valine, phenylalanine)	✓	
1048	Proteins		✓
1260	Amide III (Proteins), lipids	✓	
1297	Amide III (proteins), lipids	✓	
1338	Proteins, nucleic acids		✓
1452	Phospholipids, proteins		✓
1512	Nucleic acids	✓	
1645	Amide I, α-helix (proteins), Lipids		✓

b

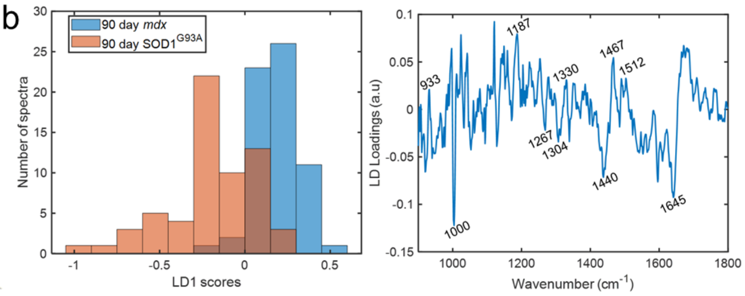

Wn. (cm-1)	Tentative Assignment	Group	
		SOD1	*mdx*
933	Proteins, α-helix		✓
1000	Proteins (phenylalanine)	✓	
1121	Proteins, Lipids		✓
1267	Amide III, α-helix (Proteins), Lipids	✓	
1304	Amide III, α-helix (Proteins), Lipids	✓	
1330	Proteins, Nucleic acids		✓
1440	Phospholipids, Proteins	✓	
1467	Lipids		✓
1512	Nucleic acids		✓
1645	Amide I, α-helix (Proteins), Lipids	✓	

Supplementary figure 6. Linear discriminant function histograms and loadings plots for SOD1^{G93A} vs. *mdx* comparisons.

(a). LD histogram, associated loadings plot and tentative peak assignments for the comparison between 30 day *mdx* and SOD1^{G93A} mice.

(b). LD histogram, associated loadings plot and tentative peak assignments for the comparison between 90 day *mdx* and SOD^{1G93A} mice.

See supplemental table one for tentative peak assignments and references.

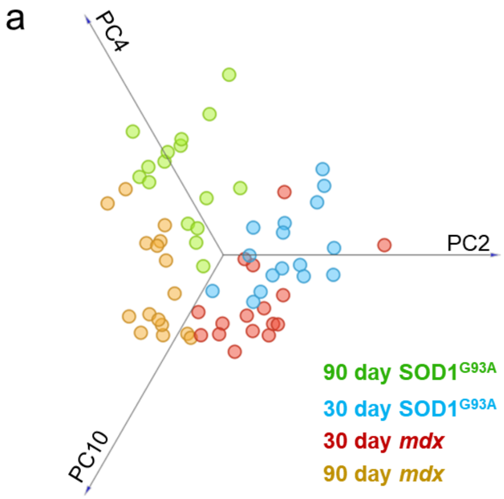

b

	4 group model			
Chemometric technique	Sens.	Spec.	Acc.	AUROC
PCA-*k*nn	0.83	0.94	0.83	0.93
**PCA-SVM**	**0.84**	**0.95**	**0.84**	**0.94**

Supplemental figure 7. Supervised four group model analysis.

(a). PCA plot with the four disease groups.

(b). Classification results for the four group model utilising (k-nn) and (SVM) algorithms. The best performing technique is shown in bold.

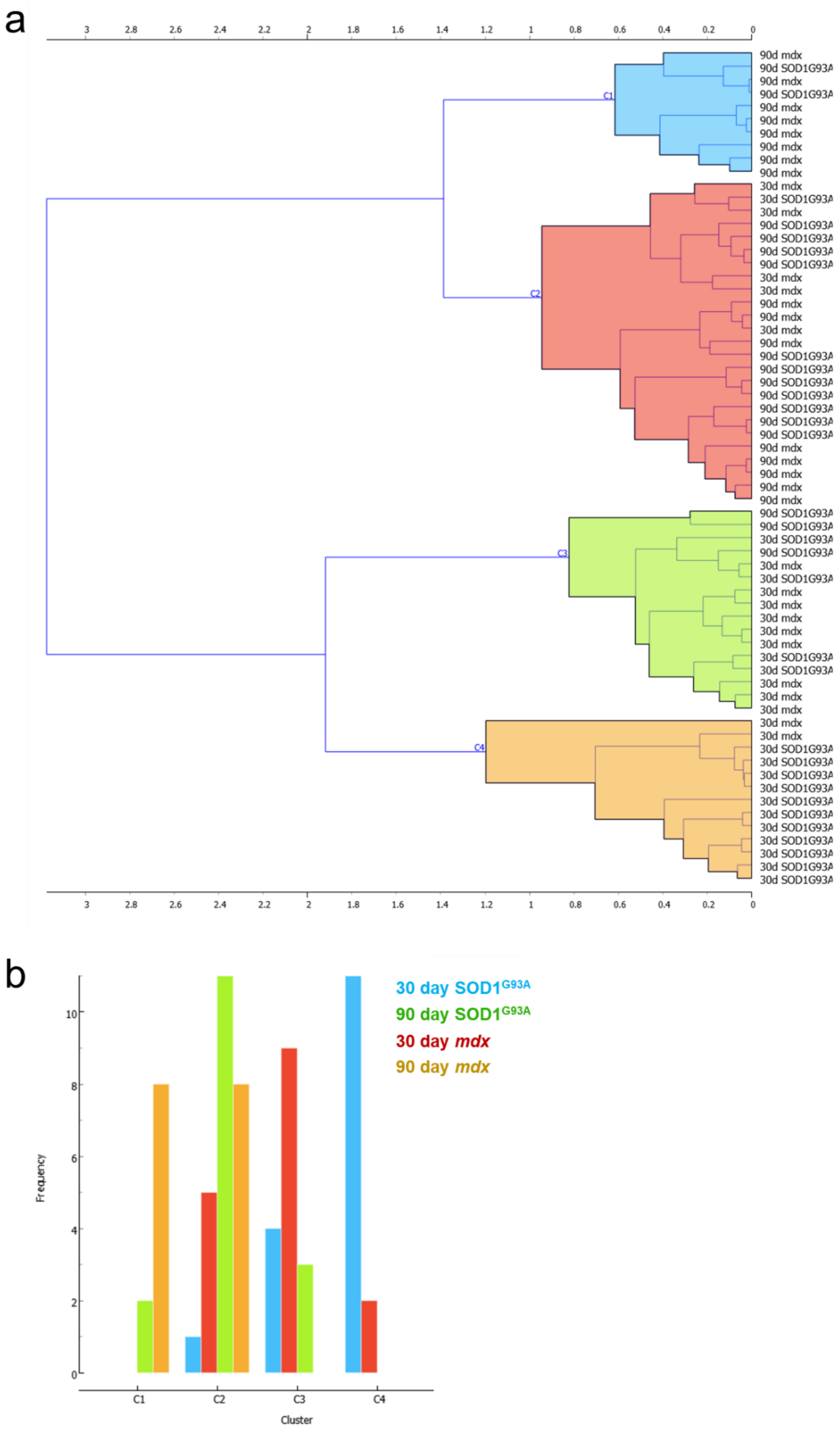

Supplemental figure 8. Unsupervised four group model analysis.

(a). PCA fed hierarchical cluster Dendrogram.

(b). Frequency distribution of different disease groups within the four clusters. d = day.

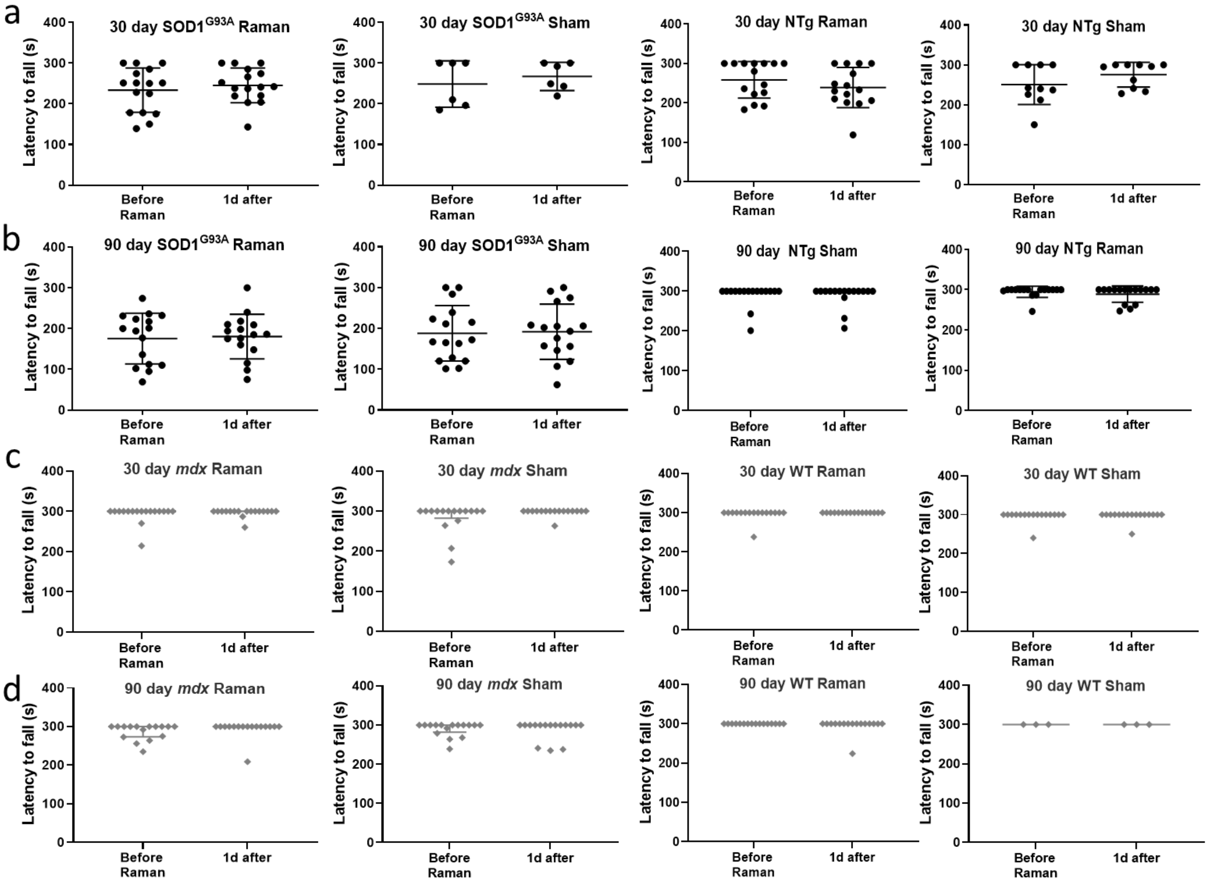

Supplementary figure 9. SOD1^{G93A}, *mdx* and respective control rotarod performance 1-day post-*in vivo* Raman spectroscopy or sham procedure.

(a). Rotarod performance of 30 day SOD1^{G93A} mice and non-transgenic littermate control mice.

(b). Rotarod performance of 90 day SOD1^{G93A} mice and non-transgenic littermate control mice. Note that by 90 days the non-transgenic mice performance much better with most mice completing the assessment.

(c). Rotarod performance of 30 day *mdx* mice and WT control mice.

(d). Rotarod performance of 90 day old *mdx* mice WT control mice.

No significant differences were observed between the pre-procedure and post-procedure performances.

A subset of these mice also underwent further testing at two weeks post-procedure; the data from these mice are shown in the main body of the paper.

a

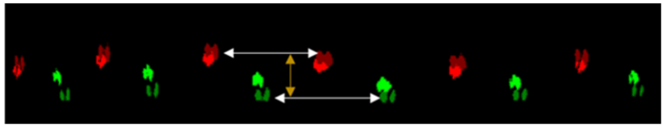

b

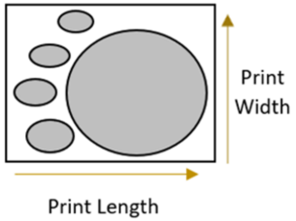

c

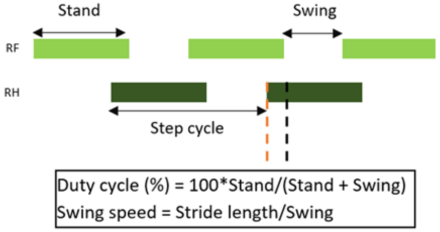

d

	30-day $SOD1^{G93A}$	30-day C57Bl/6	30-day *mdx*	30-day WT C57Bl/10	90-day $SOD1^{G93A}$	90-day NTg C57Bl/6	90-day *mdx*	90-day WT C57Bl/10
**Duration**	NS	NS	NS	NS	NS	NS	NS	NS
**Step pattern #**	NS	NS	NS	NS	NS	NS	NS	NS
**Step pattern Ca**	NS	NS	NS	NS	NS	NS	NS	NS
**Step pattern Cb**	NS	NS	NS	NS	NS	NS	NS	NS
**Step pattern Aa**	NS	NS	NS	NS	NS	NS	NS	NS
**Step pattern Ab**	NS	NS	NS	NS	NS	NS	NS	NS
**Regularity index**	NS	NS	NS	NS	NS	NS	NS	NS
**BOS - front**	NS	NS	*0.02 (d)*	NS	NS	NS	NS	NS
**BOS - hind**	NS	NS	NS	NS	NS	NS	NS	NS
**Print position - right**	NS	NS	NS	NS	NS	NS	NS	NS
**Print position - left**	NS	NS	NS	NS	NS	NS	NS	NS
**Support - zero**	NS	NS	NS	NS	NS	NS	NS	NS
**Support - single**	NS	NS	NS	NS	NS	NS	NS	NS
**Support - diagonal**	NS	NS	NS	NS	NS	NS	NS	*0.005(i)*
**Support - lateral**	NS	NS	NS	NS	NS	NS	NS	NS
**Support - gridle**	NS	NS	NS	NS	NS	NS	NS	NS
**Support - three**	NS	NS	NS	NS	NS	NS	NS	NS
**Support - four**	NS	NS	NS	NS	NS	NS	NS	NS
**Initial contact - RF/LH**	NS	NS	NS	NS	NS	NS	NS	NS
**Initial contact - LF/RH**	NS	NS	NS	NS	NS	NS	NS	NS
**Max contact - RF/LH**	NS	NS	NS	NS	NS	NS	NS	NS
**Max contact - LF/RH**	NS	NS	NS	NS	NS	NS	NS	NS
**Max area - RF/LH**	NS	NS	NS	NS	NS	NS	NS	NS
**Max area - LF/RH**	NS	NS	NS	NS	NS	NS	NS	NS
**Intensity - RF/LH**	NS	NS	NS	NS	NS	NS	NS	NS
**Intensity - LF/RH**	NS	NS	NS	NS	NS	NS	NS	NS
**Print width - RF/LH**	NS	NS	NS	NS	NS	NS	NS	NS
**Print width - LF/RH**	NS	NS	NS	NS	NS	NS	NS	NS
**Print length - RF/LH**	NS	NS	NS	NS	NS	NS	NS	NS
**Print length - LF/RH**	NS	NS	NS	NS	NS	NS	NS	NS
**Print area - RF/LH**	NS	NS	NS	NS	NS	NS	NS	NS
**Print area - LF/RH**	NS	NS	NS	NS	NS	NS	NS	NS
**Stand time - RF/LH**	NS	NS	NS	NS	NS	NS	NS	NS
**Stand time - LF/RH**	NS	NS	NS	NS	NS	NS	NS	NS
**Swing - RF/LH**	NS	NS	NS	NS	NS	NS	NS	NS
**Swing - LF/RH**	NS	NS	NS	NS	NS	NS	NS	NS
**Stride length - RF/LH**	NS	NS	NS	NS	NS	NS	NS	NS
**Stride length - LF/RH**	NS	NS	NS	NS	NS	NS	NS	NS
**Duty cycle - RF/LH**	NS	NS	NS	NS	NS	NS	NS	NS
**Duty cycle - LF/RH**	NS	NS	*0.02 (d)*	NS	NS	NS	NS	NS
**Swing speed - RF/LH**	NS	NS	NS	NS	NS	NS	NS	NS
**Swing speed - LF/RH**	NS	NS	NS	NS	NS	NS	NS	NS
**Stand index - RF/LH**	NS	NS	NS	NS	NS	NS	NS	NS
**Stand index - LF/RH**	NS	NS	NS	NS	NS	NS	NS	NS

e

	30-day $SOD1^{G93A}$	30-day NTg C57Bl/6	30-day *mdx*	30-day WT C57Bl/10	90-day $SOD1^{G93A}$	90-day NTg C57Bl/6	90-day *mdx*	90-day WT C57Bl/10
**Duration**	NS	NS	NS	NS	NS	NS	NS	NS
**Step pattern Ca**	NS	NS	NS	NS	NS	NS	NS	NS
**Step pattern Cb**	NS	NS	NS	NS	NS	NS	NS	NS
**Step pattern Aa**	NS	NS	NS	NS	NS	NS	NS	NS
**Step pattern Ab**	NS	NS	NS	NS	NS	NS	NS	NS
**Regularity index**	NS	NS	NS	NS	NS	NS	NS	NS
**BOS - front**	NS	NS	NS	NS	NS	NS	NS	NS
**BOS - hind**	NS	NS	NS	NS	NS	NS	NS	NS
**Print position - right**	NS	NS	NS	NS	NS	NS	NS	NS
**Print position - left**	NS	NS	NS	NS	NS	NS	NS	NS
**Support - zero**	NS	NS	NS	NS	NS	NS	NS	NS
**Support - single**	NS	NS	NS	NS	NS	NS	NS	NS
**Support - diagonal**	NS	NS	NS	NS	NS	NS	NS	*0.021 (i)*
**Support - lateral**	NS	NS	NS	NS	NS	NS	NS	NS
**Support - gridle**	NS	NS	NS	NS	NS	NS	NS	NS
**Support - three**	NS	NS	NS	NS	NS	NS	*0.004 (d)*	NS
**Support - four**	NS	NS	NS	NS	NS	NS	NS	NS
**Initial contact - RF/LH**	NS	NS	NS	NS	NS	NS	NS	NS
**Initial contact - LF/RH**	NS	NS	NS	NS	NS	NS	NS	NS
**Max contact - RF/LH**	NS	NS	NS	NS	NS	NS	NS	NS
**Max contact - LF/RH**	NS	NS	NS	NS	NS	NS	NS	NS
**Max area - RF/LH**	NS	NS	NS	NS	NS	NS	NS	NS
**Max area - LF/RH**	NS	NS	NS	NS	NS	NS	NS	NS
**Intensity - RF/LH**	NS	NS	NS	NS	NS	NS	NS	NS
**Intensity - LF/RH**	NS	NS	NS	NS	NS	NS	NS	NS
**Print width - RF/LH**	NS	NS	NS	NS	NS	NS	NS	NS
**Print width - LF/RH**	NS	NS	NS	NS	NS	NS	NS	NS
**Print length - RF/LH**	NS	NS	NS	NS	NS	NS	NS	NS
**Print length - LF/RH**	NS	NS	NS	NS	NS	NS	NS	NS
**Print area - RF/LH**	NS	NS	NS	NS	NS	NS	NS	NS
**Print area - LF/RH**	NS	NS	NS	NS	NS	NS	NS	NS
**Stand time - RF/LH**	NS	NS	NS	NS	NS	NS	NS	NS
**Stand time - LF/RH**	NS	NS	NS	NS	NS	NS	NS	NS
**Swing - RF/LH**	NS	NS	NS	NS	NS	NS	NS	NS
**Swing - LF/RH**	NS	NS	NS	NS	NS	NS	NS	NS
**Stride length - RF/LH**	NS	NS	NS	NS	NS	NS	NS	NS
**Stride length - LF/RH**	NS	NS	NS	NS	NS	NS	NS	NS
**Duty cycle - RF/LH**	NS	NS	NS	NS	NS	NS	NS	NS
**Duty cycle - LF/RH**	NS	NS	NS	NS	NS	NS	NS	NS
**Swing speed - RF/LH**	NS	NS	NS	NS	NS	NS	NS	NS
**Swing speed - LF/RH**	NS	NS	NS	NS	NS	NS	NS	NS
**Stand index - RF/LH**	NS	NS	NS	NS	NS	NS	NS	NS
**Stand index - LF/RH**	NS	NS	NS	NS	NS	NS	NS	NS

Supplementary figure 10. Catwalk gait analysis.

(a). Example of paw prints and analysis of stride length for left and right hind paws (white arrows) and hindlimb base of support (yellow arrow).

(b). Schematic of print width, length and print area (shadowed area).

(c). Schematic of stand and swing measurements and step cycle. The initial contact (orange dashed line) and max contact measurements are shown for the second placement of the right hind paw. Formulas for the calculation of the duty cycle and swing speed displayed.

(d). Comparison between gait parameters determined from measurements made one week before and one day after the in vivo Raman procedure using the Catwalk system.

(e). Comparison between gait parameters determined one week before and two weeks after the Raman experimental procedure.

Adjusted P-values (q-values) following FDR correction ($Q=0.05$) are shown when significant ($q<0.05$). (d): decreased with time; (i): increased with time.

a

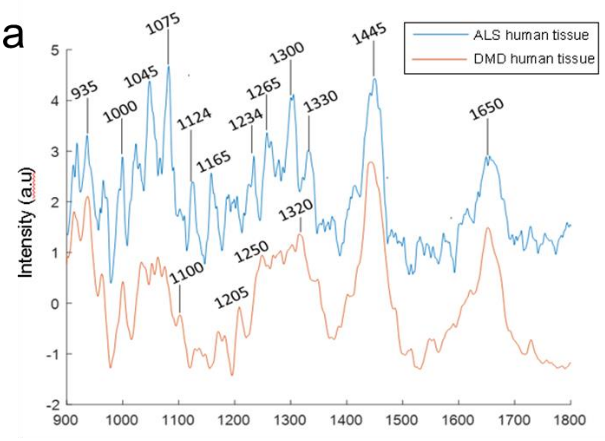

b

		Group	
Wn. (cm^{-1})	Tentative Assignment	MND	DMD
935	Proteins, α-helix	✓	✓
1000	Proteins (phenylalanine)	✓	✓
1045	Proteins (proline)	✓	
1075	Proteins (tryptophan), lipids	✓	
1100	Proteins, lipids		✓
1124	Proteins, lipids	✓	
1165	Proteins (Tyrosine, Phenylalanine)	✓	
1205	Proteins (collagen), (Phenylalanine, Tyrosine, Hydroxyproline)		✓
1234	Amide III (β-sheet proteins)	✓	
1265	Amide III (a-helix proteins), Lipids	✓	
1300	Amide III (a-helix proteins), Lipids	✓	
1320	Proteins, nucleic acids		✓
1330	Proteins, nucleic acids	✓	
1445	Phospholipids, Proteins	✓	✓
1650	Amide I, a-helix (proteins), Lipids	✓	✓

Supplementary figure 11. Human ALS and DMD spectra and tentative peak assignments.

a). Average spectra for ALS (blue) and DMD (orange).

b). Tentative peak assignments for the prominent peaks observed in the human muscle spectra. Wn – wavenumber.

See supplemental table one for tentative peak assignments and references.

Supplemental references

1. Obuchowski NA, McClish DK. Sample size determination for diagnostic accuracy studies involving binormal ROC curve indices. *Stat Med*. **1997**;*16*(13):1529–42.

2. Mead RJ, Bennett EJ, Kennerley AJ, Sharp P, Sunyach C, Kasher P, Berwick J, Pettmann B, Battaglia G, Azzouz M, *et al*. Optimised and rapid pre-clinical screening in the SOD1(G93A) transgenic mouse model of amyotrophic lateral sclerosis (ALS). *PLoS One*. **2011**;*6*(8):e23244.

3. Demsar J, Curk T, Erjavec A, Gorup C, Hocevar T, Milutinovic M, Mozina M, Polajnar M, Toplak M, Staric A, *et al*. Orange: Data mining toolbox in Python. *J Mach Learn Res*. **2013**;*14*:2349–53.

4. Stone N, Kendall C, Smith J, Crow P, Barr H. Raman spectroscopy for identification of epithelial cancers. *Faraday Discuss*. **2004**;*126*(1):141–57.

5. Bonnier F, Byrne HJ. Understanding the molecular information contained in principal component analysis of vibrational spectra of biological systems. *Analyst*. **2012**;*137*(2):322–32.

6. Frank CJ, McCreery RL, Redd DCB. Raman spectroscopy of normal and diseased human breast tissues. *Anal Chem*. **1995**;*67*(5):777–83.

7. Mahadevan-Jansen A, Mitchell MF, Ramanujam N, Malpica A, Thomsen S, Utzinger U, Richards-Kortum R. Near-infrared Raman spectroscopy for in vitro detection of cervical precancers. *Photochem Photobiol*. **1998**;*68*(1):123–32.

8. Gautam R, Vanga S, Madan A, Gayathri N, Nongthomba U, Umapathy S. Raman spectroscopic studies on screening of myopathies. *Anal Chem*. **2015**;*87*(4):2187–94.

9. Redd DCB, Feng ZC, Yue KT, Gansler TS. Raman spectroscopic characterization of human breast tissues: implications for breast cancer diagnosis. *Appl Spectrosc*. **1993**;*47*(6):787–91.

10. Puppels GJ, Olminkhof JH, Segers-Nolten GM, Otto C, de Mul FF, Greve J. Laser irradiation and Raman spectroscopy of single living cells and chromosomes: sample degradation occurs with 514.5 nm but not with 660 nm laser light. *Exp Cell Res*. **1991**;*195*(2):361–7.

11. Cheng W-T, Liu M-T, Liu H-N, Lin S-Y. Micro-Raman spectroscopy used to identify and grade human skin pilomatrixoma. *Microsc Res Tech*. **2005**;*68*(2):75–9.

12. Stone N, Kendall C, Shepherd N, Crow P, Barr H. Near-infrared Raman spectroscopy for the classification of epithelial pre-cancers and cancers. *J Raman Spectrosc*. **2002**;*33*(7):564–73.

13. Wang H, Huang N, Zhao J, Lui H, Korbelik M, Zeng H. Depth-resolved in vivo micro-Raman spectroscopy of a murine skin tumor model reveals cancer-specific spectral biomarkers. *J Raman Spectrosc*. **2011**;*42*(2):160–6.

14. Nguyen TT, Gobinet C, Feru J, -Pasco SB, Manfait M, Piot O. Characterization of type I and IV collagens by Raman microspectroscopy: Identification of spectral markers of

the dermo-epidermal junction. *Spectrosc An Int J*. **2012**;*27*(5–6):421–7.

15. Al-Rifai R, Tournois C, Kheirallah S, Bouland N, Poitevin G, Nguyen P, Beljebbar A. Subcutaneous and transcutaneous monitoring of murine hindlimb ischemia by in vivo Raman spectroscopy. *Analyst*. **2019**;*144*(15):4677–86.

16. Carew E, Asher I, Stanley H. Laser raman spectroscopy--new probe of myosin substructure. *Science*. **1975**;*188*(4191):933–6.

17. Frushour BG, Koenig JL. Raman spectroscopic study of tropomyosin denaturation. *Biopolymers*. **1974**;*13*(9):1809–19.

18. Pézolet M, Pigeon-Gosselin M, Nadeau J, Caillé JP. Laser Raman scattering. A molecular probe of the contractile state of intact single muscle fibers. *Biophys J*. **1980**;*31*(1):1–8.

19. Gniadecka M, Wulf HC, Mortensen NN, Nielsen OF, Christensen DH. Diagnosis of basal cell carcinoma by Raman spectroscopy. *J Raman Spectrosc*. **1997**;*28*(2–3):125–9.

20. Jyothi Lakshmi R, Kartha VB, Murali Krishna C, R Solomon JG, Ullas G, Uma Devi P. Tissue Raman spectroscopy for the study of radiation damage: brain irradiation of mice. *Radiat Res*. **2002**;*157*(2):175–82.

21. De Gelder J, De Gussem K, Vandenabeele P, Moens L. Reference database of Raman spectra of biological molecules. *J Raman Spectrosc*. **2007**;*38*(9):1133–47.

22. Zhu G, Zhu X, Fan Q, Wan X. Raman spectra of amino acids and their aqueous solutions. *Spectrochim Acta A Mol Biomol Spectrosc*. **2011**;*78*(3):1187–95.

23. Manoharan R, Baraga JJ, Feld MS, Rava RP. Quantitative histochemical analysis of human artery using Raman spectroscopy. *J Photochem Photobiol B Biol*. **1992**;*16*(2):211–33.

24. Mizuno A, Kitajima H, Kawauchi K, Muraishi S, Ozaki Y. Near-infrared Fourier transform Raman spectroscopic study of human brain tissues and tumours. *J Raman Spectrosc*. **1994**;*25*(1):25–9.

25. Mahadevan-Jansen A, Richards-Kortum R. Raman spectroscopy for cancer detection: a review. In: *Proceedings of the 19th Annual International Conference of the IEEE Engineering in Medicine and Biology Society. IEEE*; **1997**. p. 2722–8.

26. Malini R, Venkatakrishna K, Kurien J, Pai KM, Rao L, Kartha VB, Krishna CM. Discrimination of normal, inflammatory, premalignant, and malignant oral tissue: a Raman spectroscopy study. *Biopolymers*. **2006**;*81*(3):179–93.

27. Hartman KA, Clayton N, Thomas GJ. Studies of virus structure by Raman spectroscopy I. R17 virus and R17 RNA. *Biochem Biophys Res Commun*. **1973**;*50*(3):942–9.

28. Huang N, Short M, Zhao J, Wang H, Lui H, Korbelik M, Zeng H. Full range characterization of the Raman spectra of organs in a murine model. *Opt Express*. **2011**;*19*(23):22892–909.

29. Huang Z, McWilliams A, Lui H, McLean DI, Lam S, Zeng H. Near-infrared Raman spectroscopy for optical diagnosis of lung cancer. *Int J cancer*. **2003**;*107*(6):1047–52.

30. Kamemoto LE, Misra AK, Sharma SK, Goodman MT, Luk H, Dykes AC, Acosta T. Near-infrared micro-Raman spectroscopy for in vitro detection of cervical cancer. *Appl Spectrosc*. **2010**;*64*(3):255–61.

31. Huang Z, Bergholt MS, Zheng W, Lin K, Ho KY, Teh M, Yeoh KG. In vivo early diagnosis of gastric dysplasia using narrow-band image-guided Raman endoscopy. *J Biomed Opt*. **2010**;*15*(3):37017.

32. Minamikawa T, Harada Y, Takamatsu T. Ex vivo peripheral nerve detection of rats by spontaneous Raman spectroscopy. *Sci Rep*. **2015**;*5*(1):17165.

33. Chan JW, Taylor DS, Zwerdling T, Lane SM, Ihara K, Huser T. Micro-Raman spectroscopy detects individual neoplastic and normal hematopoietic cells. *Biophys J*. **2006**;*90*(2):648–56.

34. Frank CJ, Redd DCB, Gansler TS, McCreery RL. Characterization of human breast biopsy specimens with near-IR Raman spectroscopy. *Anal Chem*. **1994**;*66*(3):319–26.

35. Copeland RA, Spiro TG. Ultraviolet resonance Raman spectra of cytochrome c conformational states. *Biochemistry*. **1985**;*24*(18):4960–8.

36. Silveira L, Sathaiah S, Zângaro RA, Pacheco MTT, Chavantes MC, Pasqualucci CAG. Correlation between near-infrared Raman spectroscopy and the histopathological analysis of atherosclerosis in human coronary arteries. *Lasers Surg Med*. **2002**;*30*(4):290–7.

37. Chen Y, Dai J, Zhou X, Liu Y, Zhang W, Peng G. Raman spectroscopy analysis of the biochemical characteristics of molecules associated with the malignant transformation of gastric mucosa. *PLoS One*. **2014**;*9*(4):e93906.

38. Weng Y-M, Weng R-H, Tzeng C-Y, Chen W. Structural analysis of triacylglycerols and edible oils by near-infrared Fourier transform Raman spectroscopy. *Appl Spectrosc*. **2003**;*57*(4):413–8.

39. Keller S, Schrader B, Hoffmann A, Schrader W, Metz K, Rehlaender A, Pahnke J, Ruwe M, Budach W. Application of near-infrared-Fourier transform Raman spectroscopy in medical research. *J Raman Spectrosc*. **1994**;*25*(7–8):663–71.

40. Pézolet M, Pigeon M, Ménard D, Caillé JP. Raman spectroscopy of cytoplasmic muscle fiber proteins. Orientational order. *Biophys J*. **1988**;*53*(3):319–25.

41. Sato H, Maeda Y, Ishigaki M, Andriana BB. Biomedical Applications of Raman Spectroscopy. In: *Encyclopedia of Analytical Chemistry*. Chichester, UK: John Wiley & Sons, Ltd; **2014**. p. 1–12.

42. Hanlon EB, Manoharan R, Koo TW, Shafer KE, Motz JT, Fitzmaurice M, Kramer JR, Itzkan I, Dasari RR, Feld MS. Prospects for in vivo Raman spectroscopy. *Phys Med Biol*. **2000**;*45*(2):R1-59.

43. Koljenović S, Schut TB, Vincent A, Kros JM, Puppels GJ. Detection of meningioma in dura mater by Raman spectroscopy. *Anal Chem*. **2005**;*77*(24):7958–65.

44. Surmacki JM, Ansel-Bollepalli L, Pischiutta F, Zanier ER, Ercole A, Bohndiek SE. Label-

free monitoring of tissue biochemistry following traumatic brain injury using Raman spectroscopy. *Analyst*. **2017**;*142*(1):132–9.

45. Chowdary MVP, Kalyan Kumar K, Mathew S, Rao L, Krishna CM, Kurien J. Biochemical correlation of Raman spectra of normal, benign and malignant breast tissues: a spectral deconvolution study. *Biopolymers*. **2009**;*91*(7):539–46.

46. Liu C-H, Das BB, Glassman WLS, Tang GC, Yoo KM, Zhu HR, Akins DL, Lubicz SS, Cleary J, Prudente R, *et al*. Raman, fluorescence, and time-resolved light scattering as optical diagnostic techniques to separate diseased and normal biomedical media. *J Photochem Photobiol B Biol*. **1992**;*16*(2):187–209.

47. Ruiz-Chica AJ, Medina MA, Sánchez-Jiménez F, Ramírez FJ. Characterization by Raman spectroscopy of conformational changes on guanine–cytosine and adenine–thymine oligonucleotides induced by aminooxy analogues of spermidine. *J Raman Spectrosc*. **2004**;*35*(2):93–100.

48. Lykina AA, Artemyev DN, Kukushkin VI, Bratchenko IA, Aleksandrov NS, Zakharov VP. Raman spectroscopy for kidney tissue and its neoplasms research. *J Phys Conf Ser*. **2018**;*1096*(1):12116.

49. Krafft C, Neudert L, Simat T, Salzer R. Near infrared Raman spectra of human brain lipids. *Spectrochim Acta A Mol Biomol Spectrosc*. 2005;*61*(7):1529–35.

50. Lau DP, Huang Z, Lui H, Anderson DW, Berean K, Morrison MD, Shen L, Zeng H. Raman spectroscopy for optical diagnosis in the larynx: preliminary findings. *Lasers Surg Med*. **2005**;*37*(3):192–200.

51. Manoharan R, Shafer K, Perelman L, Wu J, Chen K, Deinum G, Fitzmaurice M, Myles J, Crowe J, Dasari RR, *et al*. Raman spectroscopy and fluorescence photon migration for breast cancer diagnosis and imaging. *Photochem Photobiol*. **1998**;*67*(1):15–22.

52. Rau J V., Graziani V, Fosca M, Taffon C, Rocchia M, Crucitti P, Pozzilli P, Onetti Muda A, Caricato M, Crescenzi A. RAMAN spectroscopy imaging improves the diagnosis of papillary thyroid carcinoma. *Sci Rep*. **2016**;*6*(September):35117.

53. Ó Faoláin E, Hunter MB, Byrne JM, Kelehan P, McNamara M, Byrne HJ, Lyng FM. A study examining the effects of tissue processing on human tissue sections using vibrational spectroscopy. *Vib Spectrosc*. **2005**;*38*(1–2):121–7.

54. Kaminaka S, Yamazaki H, Ito T, Kohda E, Hamaguchi H. Near-infrared Raman spectroscopy of human lung tissues: possibility of molecular-level cancer diagnosis. *J Raman Spectrosc*. **2001**;*32*(2):139–41.

55. Kishp K, Noda H. Laser Raman studies on myosin, C-protein, and myosin-C-protein Complex. *J Biochem*. **1983**;*94*(2):353–9.

56. Huang Z, Teh SK, Zheng W, Lin K, Ho KY, Teh M, Yeoh KG. In vivo detection of epithelial neoplasia in the stomach using image-guided Raman endoscopy. *Biosens Bioelectron*. **2010**;*26*(2):383–9.

57. Cárcamo JJ, Aliaga AE, Clavijo E, Brañes M, Campos-Vallette MM. Raman and surface-enhanced Raman scattering in the study of human rotator cuff tissues after shock

wave treatment. *J Raman Spectrosc*. **2012**;*43*(2):248–54.
